# Impacts 2 years after a scalable early childhood development intervention to increase psychosocial stimulation in the home: A follow-up of a cluster randomised controlled trial in Colombia

**DOI:** 10.1371/journal.pmed.1002556

**Published:** 2018-04-24

**Authors:** Alison Andrew, Orazio Attanasio, Emla Fitzsimons, Sally Grantham-McGregor, Costas Meghir, Marta Rubio-Codina

**Affiliations:** 1 Institute for Fiscal Studies, London, United Kingdom; 2 Department of Economics, University College London, London, United Kingdom; 3 Institute of Education, University College London, London, United Kingdom; 4 Institute of Child Health, University College London, London, United Kingdom; 5 Department of Economics, Yale University, New Haven, Connecticut, United States of America; 6 Inter-American Development Bank, Washington, District of Columbia, United States of America; The Hospital for Sick Children, CANADA

## Abstract

**Background:**

Poor early childhood development (ECD) in low- and middle-income countries is a major concern. There are calls to universalise access to ECD interventions through integrating them into existing government services but little evidence on the medium- or long-term effects of such scalable models. We previously showed that a psychosocial stimulation (PS) intervention integrated into a cash transfer programme improved Colombian children’s cognition, receptive language, and home stimulation. In this follow-up study, we assessed the medium-term impacts of the intervention, 2 years after it ended, on children’s cognition, language, school readiness, executive function, and behaviour.

**Methods and findings:**

Study participants were 1,419 children aged 12–24 months at baseline from beneficiary households of the cash transfer programme, living in 96 Colombian towns. The original cluster randomised controlled trial (2009–2011) randomly allocated the towns to control (*N =* 24, *n =* 349), PS (*N =* 24, *n =* 357), multiple micronutrient (MN) supplementation (*N =* 24, *n =* 354), and combined PS and MN (*N =* 24, *n =* 359). Interventions lasted 18 months. In this study (26 September 2013 to 11 January 2014), we assessed impacts on cognition, language, school readiness, executive function, and behaviour 2 years after intervention, at ages 4.5–5.5 years. Testers, but not participants, were blinded to treatment allocation. Analysis was on an intent-to-treat basis. We reassessed 88.5% of the children in the original study (*n =* 1,256). Factor analysis of test scores yielded 2 factors: cognitive (cognition, language, school readiness, executive function) and behavioural. We found no effect of the interventions after 2 years on the cognitive factor (PS: −0.031 SD, 95% CI −0.229–0.167; MN: −0.042 SD, 95% CI −0.249–0.164; PS and MN: −0.111 SD, 95% CI −0.311–0.089), the behavioural factor (PS: 0.013 SD, 95% CI −0.172–0.198; MN: 0.071 SD, 95% CI −0.115–0.258; PS and MN: 0.062 SD, 95% CI −0.115–0.239), or home stimulation. Study limitations include that behavioural development was measured through maternal report and that very small effects may have been missed, despite the large sample size.

**Conclusions:**

We found no evidence that a scalable PS intervention benefited children’s development 2 years after it ended. It is possible that the initial effects on child development were too small to be sustained or that the lack of continued impact on home stimulation contributed to fade out. Both are likely related to compromises in implementation when going to scale and suggest one should not extrapolate from medium-term effects of small efficacy trials to scalable interventions. Understanding the salient differences between small efficacy trials and scaled-up versions will be key to making ECD interventions effective tools for policymakers.

**Trial registration:**

ISRCTN18991160

## Abstract 2

In this follow up from a cluster randomized controlled trial in Colombia, Alison Andrew and colleagues investigate whether a home stimulation intervention had sustained impact on child development two years after the intervention.

## Author summary

**Why was this study done?:**

- Small-scale, high-quality programmes designed to improve parenting practices and the psychosocial stimulation that very young children experience at home can profoundly benefit children’s development and have long-term benefits to education, wages, and well-being.
- In this study we consider whether such programmes are effective at producing sustained benefits to child development when implemented at a bigger scale and using the institutional infrastructure of existing government services.

**What did the researchers do and find?:**

- We estimated the medium-term effects on child development of a scalable home stimulation intervention in Colombia by re-enrolling, 2 years after intervention activities ended, participants of a large cluster randomised controlled trial and assessing their development over multiple domains: cognition, language, school readiness, executive functioning, and behaviour.
- We found no evidence that the stimulation intervention had had any sustained impact on children’s development after 2 years. This was despite finding medium-to-small benefits immediately after the intervention finished.

**What do these findings mean?:**

- There is no guarantee that small-scale tightly controlled programmes that generate large sustained benefits to child development will continue to be effective when scaled up.
- We suggest that careful study of processes involved in running such interventions at scale and through government institutions is essential to maintain the quality of the original model. In particular, providing rigorous training, supervision, and coaching to programme staff when operating at scale is an important challenge.

## Introduction

There is considerable evidence that interventions aimed at improving maternal–child interactions and stimulation in the home benefit young children’s cognitive, language, and behavioural development in the short term, both in high-income and in low- and middle-income countries (LMICs) [1–6]. A few follow-up studies, mainly from the US, have shown sustained benefits to cognition, social behaviour, school attainment, and earnings [7]. It is these medium- and longer-term effects that are used to justify the use of public resources on policies targeting the home environments of young disadvantaged children, on the grounds that these policies pay for themselves over the long run and help to break the intergenerational transmission of poverty [6]. Such arguments are particularly pertinent in LMICs, where the number of disadvantaged children is higher and the degree of disadvantage more severe. In this context, there have recently been calls, from high-profile policymakers and academics alike, for large-scale programmes that integrate early childhood development (ECD) interventions into other services such as health, nutrition, and cash transfer programmes [8,9]. However, there is little evidence on the medium- or longer-term effects of integrated ECD interventions delivered at scale in LMICs.

In a review of 9 recent systematic and non-systematic reviews [1–8,10], we identified 5 published randomised studies of psychosocial stimulation (PS) interventions that measured effects beyond the end of intervention activities in LMICs. Of these, 4 were small-scale efficacy trials of interventions not integrated into government services. Four of the 5 were home-visiting interventions. A Jamaican study found medium- and long-term benefits from a PS intervention delivered through weekly home visits for 2 years, beginning when children, all stunted, were 9–24 months old. By age 22 years, the intervention group had higher IQ, higher educational attainment, less violent behaviour, less depression, and higher earnings [1,11,12]. Another Jamaican study using a similar intervention with low birth weight children born at term found moderate effects on cognition and behaviour at age 6 years, 4 years after the intervention ended [13]. An early centre-based intervention in Colombia found that the initial cognitive benefits were reduced but remained significant 2 years after the intervention ended, at age 8 or 9 years [14]. A South African home-visiting intervention delivered from pregnancy until 6 months postpartum improved children’s attachment, but the small effect on mental development was not significant (*p =* 0.094) at 18 months [15,16]. The fifth study was the only larger-scale stimulation intervention integrated into a government service that reported medium-term effects [17]. Set in rural Pakistan, the study found benefits to cognition, executive function, pre-academic skills, and behaviour from monthly group sessions and home visits 2 years after the intervention ended, when children were 4 years of age [17]. However, there are concerns about the study design in that there were de facto just 4 units of randomisation [18]. There is therefore an urgent need for medium- and longer-term follow-ups of robust evaluations of child development interventions integrated into government services.

In 2009–2011 we conducted a 2 × 2 factorial cluster randomised controlled trial with 96 towns (clusters) and 1,419 children aged 12–24 months at enrolment, to assess the effect of a PS home-visiting intervention and of multiple micronutrient (MN) supplementation, both separately and combined, on child development in Colombia [19]. Both interventions lasted 18 months. We aimed to design an implementation model that could feasibly be used nationwide. Key to ensuring scalability was integrating delivery into existing institutions. To this end, we spread over a wide geographical area and operated both interventions through the institutional infrastructure of Colombia’s largest national welfare programme, Familias en Acción (FeA). FeA began in 2001–2002 and entitled the poorest 20% of Colombian households to monthly transfers of between US$8 and US$16 per child, conditional on children attending health checkups (for children under 7 years old) and school (for children 6–17 years old). An evaluation showed that FeA significantly increased total household consumption and school attendance for older children [20]. We worked in towns where FeA had operated since its creation and hence had been running for 7 years by the start of the home-visiting intervention. Our sampling frame was young children from FeA beneficiary households, most of whom would have still been eligible for FeA at the time of this follow-up. We employed and trained the locally elected representatives of FeA, known as Madres Líderes, as home visitors to deliver both interventions. This approach substantially reduced the intensity of supervision from that in earlier efficacy trials of the stimulation intervention, but provided a potential blueprint for scaling up the intervention nationally.

The stimulation intervention was based on a Jamaican home-visiting model that has since been adapted and made available online under the name ‘Reach Up’ (http://www.reachupandlearn.com) [21]. It consisted of weekly home visits during which the home visitor demonstrated play activities to mother and child using low-cost or homemade toys and picture books, adapted to the context. The visits followed a structured curriculum with an emphasis on cognition and language, aimed to increase and improve maternal–child interactions and the mother’s ability to promote her child’s development through play. Home visitors encouraged mothers to continue the play activities between visits and to integrate them in their daily routines. In all, 97% of those targeted received at least 1 home visit, and children on average received 63 visits [19]. Immediately after the PS intervention finished, measures showed that the intervention had improved children’s cognitive scores by 0.26 standard deviations (SD) (*p =* 0.002) and receptive language by 0.22 SD (*p =* 0.032), assessed on the Bayley Scales of Infant and Toddler Development–Third Edition (Bayley-III) [22]. There were also substantial improvements (0.53 SD, *p <* 0.001) to stimulation in the home (play activities and materials) [23].

The MN supplementation provided daily multiple MN powder containing iron, zinc, vitamin A, vitamin C, and folic acid for all children below 6 years of age in the household. The supplements were designed to reduce the prevalence of anaemia, which affects 46.6% of children under 2 years old from Colombia’s lowest socio-economic stratum [24]. We found no short-term impact of MN supplementation on child development or nutritional status [19,24].

The aim of the current study was to test whether the stimulation intervention had sustained impacts on child development 2 years after it ended and to quantify the magnitude of these effects. At the time of this follow-up, the children were 4.5–5.5 years old and soon to start primary school. Developmental abilities at primary school entrance are important because they explain much of the gap in later educational attainment between children from different socio-economic backgrounds [25]. Given the initial lack of impacts from MN supplementation, we focus the discussion on the effects of PS, although we report impacts for both interventions.

## Methods

### Study design and participants

In the initial cluster randomised controlled trial, 96 towns (clusters) were randomly allocated, in equal numbers, to 4 groups: (i) control, (ii) PS, (iii) multiple MN supplementation, and (iv) both interventions combined. Study participants were children aged 12–24 months from FeA beneficiary families living in these towns. Nationally, the poorest 20% of households are eligible for FeA, but this fraction is substantially higher in our study area. We chose towns with between 2,000 and 42,000 inhabitants where FeA had been active since it began in 2002 from 3 central regions of Colombia, comprising 7 departments: (i) Cundinamarca, Boyacá, and Santander; (ii) Antioquia, Risaralda, and Caldas; and (iii) Huila and Tolima. We selected towns similar in terms of their cultures and customs and that were relatively safe and unexposed to the ongoing conflict in Colombia. The area covered by the study is roughly 3 times the size of England. In every town, we randomly selected 3 Madres Líderes, the elected representatives of FeA beneficiaries. We listed all children aged 12–24 months from families represented by the 3 Madres Líderes through a door-to-door listing exercise and randomly chose 5 per Madre Líder for enrolment. We thus assessed 1,440 children for eligibility, of whom 11 were subsequently found to be out of the age range and a further 10 had incomplete measures of child development (Bayley-III) at baseline, leaving 1,419 study children. After 18 months of intervention activities we reassessed participant children on the Bayley-III. Further details of the study design and short-term impacts are provided elsewhere [19].

We attempted to re-enrol all 1,419 children 2 years after the end of intervention activities, when they were 4.5 to 5.5 years old. Prior to data collection, we re-contacted families by telephone to update their addresses and to motivate their continued participation in the study through entry into a prize raffle (S1 Appendix). Text messages, with no reference to the intervention or its content, were also sent to all mothers on Mother’s Day and on their child’s birthday (S1 Appendix). Follow-up assessments of child development and a household survey collecting socio-demographic information and measures of the quality of the home stimulation environment were conducted between 26 September 2013 and 11 January 2014.

### Ethics statement

All primary caregivers provided written informed consent. Ethical approval for the follow-up study was obtained from the research ethics committees of University College London, London, UK (2168/007), and Pontificia Universidad Javeriana, Bogotá, Colombia.

### Randomisation and masking

Randomisation was done at the level of the cluster (town), after stratification by region. Within each of the 3 regions, 8 towns were randomly allocated to each of the 3 treatment groups and the control group using computer-generated random numbers.

Study participants were aware of their intervention group, and we did not use a placebo for MN supplementation for practical reasons. The data collection team in the follow-up study, consisting of 6 testers and 9 interviewers, were blind to treatment allocation.

### Procedures

#### Primary outcomes

We measured a broad range of cognitive and language functions, executive function, school readiness, and behaviours. All measures are summarised in S1 Table.

We assessed cognition and language using selected subscales of the Woodcock–Muñoz (WM) Test of Cognitive Abilities and the WM Test of Achievement [26], the Spanish versions of the third edition of the Woodcock–Johnson Tests of Cognitive Abilities and Achievement. We used 6 subscales in the WM cognitive test covering concept formation, visual matching, retrieval fluency, picture recognition, decision speed, and memory for names. We measured expressive language with the picture vocabulary subscale of the WM achievement test, and receptive language with the Spanish version of the Peabody Picture Vocabulary Test–Revised, the Test de Vocabulario en Imágenes Peabody (TVIP) [27]. The TVIP and subscales of the WM cognitive and achievement tests have been used in Colombia previously [28]. We also collected measures of inhibitory control and working memory using the pencil tapping task (PTT) [29] and of pre-academic skills using a subset of age-appropriate items from the Daberon Screening for School Readiness–Second Edition (Daberon-2) [30]. Where necessary, we adapted items and translated the tests into Spanish and back-translated to English. We piloted, adapted, and translated items to ensure functional equivalence (see S2 Appendix for details). Given limited administration time and since gross motor abilities were not directly targeted by the intervention, we did not measure them.

All tests were administered directly to the child in a community centre by 1 of 6 psychology graduates (testers), after the testers completed 3 weeks training and practice. Prior to beginning field assessments, we measured inter-rater reliabilities between pairs of testers and between testers and the trainer: there was exact agreement on final scores in 85.6% of cases, across all scales. The assessment session took no more than 75 minutes allowing for 2 short breaks. As in previous rounds, clusters were organised in geographically practical routes, which were randomly assigned to testers, and 10% of assessments were observed by the supervisor to ensure testing quality.

We assessed children’s behaviour using the Spanish versions of the parental-report Strengths and Difficulties Questionnaire (SDQ) for children ages 4 to 17 years [31,32] and the attentional focusing and inhibitory control scales of the Children’s Behavior Questionnaire (CBQ) [33,34]. For both, we estimated effects by subscale. Following a suggestion from peer review and due to the lower reliability of individual subscales of the SDQ [35], we also combined items from the 4 ‘difficulty’ subscales of the SDQ to create the SDQ Total Difficulties subscale. Both the SDQ and CBQ were collected by caregiver report at home as part of the household survey.

This follow-up did not have a complete prospective analysis plan. We aimed to assess impacts on the same broad domains of child development as those outlined in the initial study protocol (S3 Appendix) and used in the short-term evaluation [19] but with a stronger focus on executive functioning, school readiness, and behavioural development, since these domains are of greater relevance at this older age. Since children were too old for the Bayley-III, the instrument used at endline in the initial study (i.e., the end of intervention delivery), we sought age- and domain-appropriate alternatives. All tests used were specified in the funding application (S4 Appendix), other than the Daberon-2 and the PTT, which were identified later and showed good performance during the piloting. Prior usage of the tests in Colombia, adequate performance in piloting, and limiting total testing time to 75 minutes, allowing breaks, to ensure the child’s concentration were all of consideration in making final decisions on which tests to use. The piloting report (S2 Appendix) documents this process and the final choice of child development measures.

#### Secondary outcomes

We measured home stimulation using 2 scales of the United Nations Children’s Fund’s family care indicators (FCIs) [36]: the variety of play activities done with adults in the previous 3 days and the variety of available play materials. Both scales were collected in the household survey and were considered secondary (intermediate) outcomes in that they could mediate the effect of the intervention on primary outcomes. Following a suggestion from peer review, we also report impacts on maternal depressive symptoms, measured using the Spanish version of the Center for Epidemiologic Studies Short Depression Scale (CES-D 10), a measure we also used at endline [19,37–39].

#### Scoring and standardisation of primary and secondary outcomes

Four of the tests (WM cognitive test subscales 6, 12, and 16 and the PTT) only recorded the number of correct responses (S1 Table). For the remaining measures, we constructed ‘raw’ scores from the item-level data using a 2-parameter item response theory (IRT) model for binary data, or a graded response model (GRM) for ordinal data. These methods convert patterns of item responses into continuous scores taking into account the estimated difficulty and discriminatory ability of each item. Several WM subscales have stopping rules by blocks of items (e.g., stop if child scores 2 points or fewer in the first 5 items). This feature implies that non-linear methods are required to correct for discontinuities in stopping rules and to obtain well-behaved distributions of scores. The WM subscales are typically scored using IRT/GRM algorithms, available from the test publisher, parameterised using estimates of item functioning from analysis of a norming sample of 1,413 Spanish-speaking children from the US, 6 Latin American countries, and Spain [26]. We followed an IRT/GRM approach but used difficulty and discriminatory parameters estimated from our own sample (*n =* 1,255) since the functioning of items in the norming sample is likely to be different from that in our study sample. S5 Appendix provides more details.

We internally standardised child development scores for age using the age-specific mean and SD of the raw scores in the control group, estimated non-parametrically. The resulting standardised scores were thus distributed with 0 mean and unit variance in the control group.

### Statistical analysis

As planned prior to data collection and analysis, the statistical methodology followed that of the short-term evaluation [19].

Assuming an attrition rate (10.7%) and an intra-cluster correlation coefficient (0.04) equal to those at endline, we calculated the minimum detectable effect (MDE) of our study, without accounting for efficiency gains from controlling for covariates, at 80% power and for 2-tailed hypothesis tests of size (α) 0.05. Our design had a MDE of 0.27 SD for testing the mean of 1 treatment group against the control and 0.19 SD when pooling the 2 stimulation groups and the 2 groups who received no stimulation.

We assessed baseline balance in the analysis sample across key child, mother, and household characteristics by jointly testing the hypothesis that the mean of all 3 treatment groups and the control group were equal, adjusting *p*-values for clustering at the town level.

Most of our measures do not have a published validation analysis for a Colombian population. To ensure poor measurements were not hampering our analysis, we investigated the validity of our measures by estimating their mean reliability (calculated through the IRT/GRM estimation procedure)—namely, the proportion of variance due to variation in the underlying construct being measured (details in S6 Appendix)—and correlations with each other, previous measures of child development, age, and socio-economic characteristics.

As in the short-term evaluation, we used exploratory factor analysis on all measures of child development to identify underlying constructs and create summary indices. Creating summary indices of multiple outcome measures solves the multiple testing problem whereby testing multiple null hypotheses simultaneously means the probability of falsely rejecting 1 hypothesis is greater than the stated size (α) of the individual tests. Factor analysis on multiple measures of child development can also help in constructing outcome variables more fundamentally related to the underlying constructs, thus minimising measurement error. Thus, we used these summary indices as our primary outcomes. We kept and rotated all factors with an eigenvalue greater than 1 (Kaiser criterion [40]), and created summary indices using these factor loadings. We rescaled the factor indices to have 0 mean and unit variance in the control group so that effect sizes are reported relative to the SD of the control group.

Like in our analysis of endline data, all analyses were on an intention-to-treat basis. We used ordinary least squares linear regression to estimate the effect of being in each of the 3 intervention arms, relative to control, on the summary indices of child development, on each individual measure of child development, on the measures of the quality of the home environment, and on maternal depressive symptoms. As in the analysis of short-term impacts, all child development regressions controlled for standardised baseline scores on the Bayley-III. For behavioural outcomes, we additionally controlled for the baseline scores of the difficult, unadaptable, unstoppable, and unsociable scales in the Infant Characteristics Questionnaire [41]. For the 2 measures of the quality of the home environment and for maternal depressive symptoms, we controlled for baseline measures of the same scales. We controlled for sex and tester dummies (a set of binary variables indicating which tester performed the assessment) to increase precision in all analysis and clustered errors at the town level in all inference, using the White estimator extended for spatial correlation [42]. We used 2-tailed hypothesis tests throughout.

We assessed whether medium-term impacts on the cognitive factor differed significantly from short-term impacts on cognition and receptive language, as measured using Bayley-III. Bayley-III scores were age standardised and scaled to have 0 mean and unit variance in the control group (using the method described above) so short- and medium-term effect sizes are directly comparable. We tested the null hypothesis that the short-term and medium-term impacts were the same using Stata’s *suest* command to combine the short-term and medium-term estimators and estimate their joint covariance matrix (details in S7 Appendix) [43].

We used Stata version 14 for all analyses including the IRT/GRM methods and standardisation procedures. The trial was registered with the ISRCTN registry, number ISRCTN18991160.

## Results

**Fig 1:**
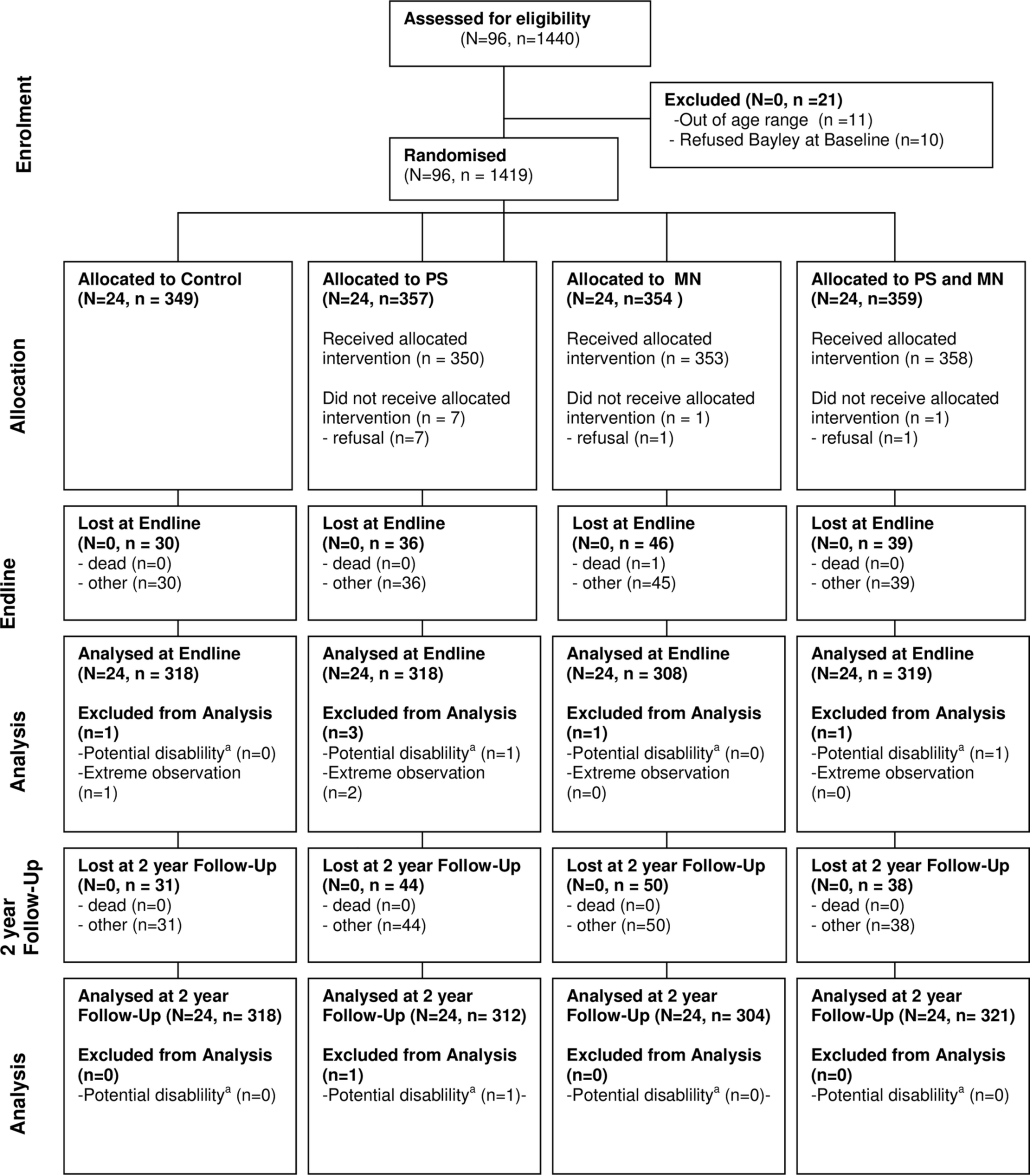
Study flow diagram. ^a^Potential disability classed as child scoring less than 3 SD below the mean on the cognitive scale of the Bayley-III at baseline, relative to external norms. Bayley-III, Bayley Scales of Infant and Toddler Development–Third Edition; MN, micronutrient; PS, psychosocial stimulation.

We measured 1,256 of the 1,419 study children with complete baseline data (Fig 1). Sample loss was 11.49% and was not significantly related to treatment status, age, or baseline child development (S2 Table). Girls were significantly more likely to be lost to follow-up than boys, but the difference was very small (the sample was 49.5% male at enrolment and 50.7% male at this follow-up) and does not introduce bias since sex is balanced across treatment status. As in the initial evaluation, we excluded 1 child with a baseline Bayley-III cognitive score less than 3 SD below the mean of the external norms, due to potential disability. The resulting 1,255 children are well balanced across the 4 groups in their characteristics, their mothers’ characteristics, and their household characteristics, measured at baseline (Table 1). At the time of this follow-up, the mean (SD) age in the analysis sample was 61.8 (3.8) months, and 51% were male.

**Table 1:** Baseline characteristics and balance for 2-year follow-up analysis sample.

Characteristic	Control (*n =* 318)	Stimulation (*n =* 312)	Supplementation (*n =* 304)	Stimulation and supplementation (*n =* 321)	*p-*Value	*n*
**Child characteristics**						
Age (months)	18.34 (4.03)	18.01 (3.77)	17.83 (3.66)	17.98 (3.71)	0.565	1,255
Birthweight (grams)	3,232.50 (554.54)	3,257.79 (480.47)	3,261.89 (492.21)	3,233.42 (526.70)	0.892	1,161
Stunted (height for age *z*-score < −2 SD)	0.17 (0.37)	0.14 (0.34)	0.11* (0.31)	0.14 (0.34)	0.308	1,232
Bayley-III raw scores						
Cognitive	52.16 (7.55)	51.44 (7.46)	51.47 (7.15)	51.89 (7.51)	0.763	1,255
Receptive Language	20.56 (5.01)	20.29 (4.99)	19.95 (5.22)	20.03 (4.80)	0.815	1,255
Expressive Language	20.52 (6.37)	20.36 (6.74)	19.73 (6.20)	19.92 (6.07)	0.723	1,255
Fine Motor	34.82 (3.96)	34.45 (4.07)	34.18 (4.00)	34.03* (3.91)	0.374	1,255
Gross Motor	50.53 (6.85)	50.88 (7.30)	50.32 (6.23)	50.32 (6.84)	0.828	1,255
**Mother characteristics**						
Age (years)	26.18 (6.99)	26.87 (6.93)	26.04 (6.20)	26.47 (6.51)	0.301	1,228
Education (years)	7.80 (3.51)	7.17* (3.46)	7.36 (3.48)	7.51 (3.47)	0.360	1,204
Married	0.70 (0.46)	0.71 (0.46)	0.70 (0.46)	0.65 (0.48)	0.326	1,228
**Household characteristics**						
Household size	5.17 (2.24)	5.32 (2.32)	5.26 (2.16)	5.19 (2.20)	0.888	1,255
Wealth index^a^	−0.07 (0.92)	0.04 (0.99)	0.04 (1.04)	0.01 (1.05)	0.698	1,255
Family care indicators (home environment)						
Types of play materials^b^	3.36 (1.59)	3.39 (1.51)	3.15 (1.57)	3.09 (1.47)	0.132	1,254
Types of play activities^c^	3.67 (1.74)	3.70 (1.71)	3.68 (1.64)	3.63 (1.67)	0.976	1,254

This table summarises baseline characteristics for the analysis sample used in this 2-year follow-up. The construction of this sample is depicted in Fig 1. Data are mean (SD). *p-*Values jointly test the hypothesis that the mean of all 4 treatment groups are equal, adjusted for clustering at the town level.

*p < 0.1: 2-tailed *p*-values for difference compared to control group.

^a^First principal component of household assets and characteristics: dirt floor, solid walls, crowding index, home ownership, sewage, and ownership of car, computer, blender, refrigerator, washing machine, and mobile phone.

^b^Toys that make or play music; toys or objects meant for stacking, constructing, or building; things for drawing, writing, colouring, and painting; toys for moving around; toys to play pretend games; picture books and drawing books for children; and toys for learning shapes and colours.

^c^Reading books or looking at picture books; telling stories to child; singing songs with child; taking child outside home place or going for a walk; playing with child with toys; spending time with child scribbling, drawing, or colouring; and spending time with child naming things or counting.

Bayley-III, Bayley Scales of Infant and Toddler Development–Third Edition.

All measures of cognition, language, and school readiness had high mean reliabilities of between 0.699 and 0.938, except concept formation (reliability = 0.371), which we therefore excluded from the analyses (Table A in S6 Appendix). The remaining tests correlated with each other, age, Bayley-III scores from endline (measured at 30–42 months), household wealth, and mother’s education in the expected direction, suggesting validity of the measures (Tables A and C in S6 Appendix). The reliabilities of the behavioural measures were generally lower (Table B in S6 Appendix), probably due to having to depend on maternal report. However, the behavioural measures correlated with each other, endline measures of behavioural development, and socio-economic characteristics in the theoretically expected directions (Tables B and C in S6 Appendix).

The exploratory factor analysis of child development measures resulted in 2 factors, with eigenvalues 4.08 and 1.68. All 9 cognitive (5 WM cognitive subscales), language (WM Expressive Language and TVIP), school readiness (Daberon-2), and executive function (PTT) measures loaded on the first factor, with all loadings above 0.4 and 5 loadings greater than 0.6. We refer to this first factor as the ‘cognitive’ factor. Measures of behaviour, with the exception of SDQ Emotional Symptoms and SDQ Peer Problems, which did not load strongly onto either factor, loaded on the second factor, which we refer to as the ‘behavioural’ factor (S3 Table). While the grouping of measures into ‘cognitive’ and ‘behavioural’ implied by the factor analysis is common in the literature, we draw readers’ attention to the fact that the ‘behavioural’ measures were collected by maternal report in the home while the ‘cognitive’ measures were directly assessed by a psychologist in the test centre, which may have contributed to this grouping. We created summary measures of each factor using the estimated factor loadings.

Table 2 reports estimates of the effect of the home stimulation intervention and MN supplementation, separately and in combination, on measures of cognition, language, school readiness, and executive function 2 years after the interventions ended. There was no evidence that either intervention affected any of the children’s individual test scores or the cognitive factor. In interpreting the effect sizes, measured in SDs of the control group, it is useful to note that the difference in cognitive scores between children whose mothers had completed high school and those who had not was 0.473 SD (0.188 SD for the behavioural factor) (S4 Table). Medium-term impacts of the stimulation intervention on the cognitive factor are significantly smaller than short-term impacts on cognition and receptive language (Table D in S7 Appendix).

**Table 2:** Estimated treatment effects on child cognition, language, school readiness, and executive functioning.

Outcome	Test/subscale	Stimulation	*p-*Value	Supplementation	*p-*Value	Stimulation and supplementation	*p-*Value	*n*
**Cognitive factor**		−0.03	0.76	−0.04	0.69	−0.11	0.27	1,243
		(−0.23 to 0.17)		(−0.25 to 0.16)		(−0.31 to 0.09)		
**Cognition**	WM Visual Matching	−0.08	0.41	−0.08	0.36	−0.02	0.83	1,251
		(−0.26 to 0.11)		(−0.26 to 0.09)		(−0.17 to 0.14)		
	WM Retrieval Fluency	−0.01	0.87	−0.01	0.88	−0.21*	0.064	1,251
		(−0.19 to 0.16)		(−0.19 to 0.16)		(−0.43 to 0.01)		
	WM Picture Recognition	0.04	0.66	0.13	0.18	0.03	0.74	1,254
		(−0.14 to 0.22)		(−0.06 to 0.32)		(−0.14 to 0.19)		
	WM Decision Speed	−0.08	0.41	−0.14	0.14	−0.05	0.63	1,252
		(−0.27 to 0.11)		(−0.33 to 0.05)		(−0.23 to 0.14)		
	WM Memory for Names	−0.09	0.36	−0.07	0.50	−0.06	0.54	1,252
		(−0.29 to 0.11)		(−0.27 to 0.13)		(−0.25 to 0.13)		
**Language**	WM Expressive Language	−0.00	0.98	−0.08	0.33	−0.13	0.16	1,255
		(−0.16 to 0.15)		(−0.26 to 0.09)		(−0.31 to 0.05)		
	TVIP (receptive language)	0.00	1.00	−0.07	0.49	−0.05	0.63	1,253
		(−0.19 to 0.19)		(−0.29 to 0.14)		(−0.26 to 0.16)		
**School readiness**	Daberon-2 (school readiness)	0.02	0.85	0.03	0.76	−0.11	0.27	1,255
		(−0.17 to 0.21)		(−0.18 to 0.24)		(−0.30 to 0.09)		
**Executive functioning**	PTT (inhibitory control and working memory)	−0.01	0.90	0.02	0.86	−0.08	0.36	1,251
		(−0.20 to 0.18)		(−0.16 to 0.19)		(−0.27 to 0.10)		

The 95% CIs (in parentheses) and *p*-values are adjusted for clustering at the town level. All scores are standardised non-parametrically with respect to age and have 0 mean and unit variance in the control group. Estimates control for baseline levels of cognitive, receptive language, expressive language, fine motor, and gross motor development, as assessed by the Bayley-III; children’s sex; and tester dummies. Scoring of all measures is outlined in S1 Table.

*p < 0.1: 2-tailed *p*-values for difference compared to control group.

Bayley-III, Bayley Scales of Infant and Toddler Development–Third Edition; Daberon-2, Daberon Screening for School Readiness–Second Edition; PTT, pencil tapping task; WM, Woodcock–Muñoz; TVIP, Test de Vocabulario en Imágenes Peabody.

Similarly, we found no evidence of any effect on the behavioural factor. We did estimate a positive and significant effect of the supplementation intervention (alone and combined with stimulation) on inhibitory control (Table 3). However, we do not consider this a robust finding: it was not echoed by effects on other measures of behavioural development and would likely no longer be significant if *p*-values were adjusted for multiple hypothesis testing.

**Table 3:** Estimated treatment effects for child behaviour.

Outcome	Test/subscale	Stimulation	*p-*Value	Supplementation	*p-*Value	Stimulation and supplementation	*p-*Value	*n*
**Behavioural factor**		0.01	0.89	0.07	0.45	0.06	0.49	1,242
		(−0.17 to 0.20)		(−0.12 to 0.26)		(−0.11 to 0.24)		
**Behaviour**	SDQ Hyperactivity^a^	−0.04	0.69	0.03	0.77	−0.01	0.96	1,249
		(−0.22 to 0.15)		(−0.17 to 0.22)		(−0.18 to 0.17)		
	SDQ Emotional Symptoms^a^	0.03	0.70	0.06	0.48	0.02	0.78	1,249
		(−0.12 to 0.17)		(−0.10 to 0.22)		(−0.14 to 0.19)		
	SDQ Conduct Problems^a^	0.01	0.93	0.02	0.86	0.02	0.86	1,249
		(−0.18 to 0.20)		(−0.16 to 0.19)		(−0.16 to 0.19)		
	SDQ Peer Problems^a^	0.00	0.98	−0.06	0.64	−0.01	0.92	1,249
		(−0.21 to 0.21)		(−0.31 to 0.19)		(−0.21 to 0.19)		
	SDQ Prosocial Behaviour	0.01	0.88	0.11	0.20	0.03	0.67	1,249
		(−0.14 to 0.16)		(−0.06 to 0.28)		(−0.11 to 0.17)		
	CBQ Attentional Focusing	−0.00	0.98	−0.02	0.81	0.01	0.94	1,249
		(−0.20 to 0.19)		(−0.21 to 0.16)		(−0.17 to 0.19)		
	CBQ Inhibitory Control	0.07	0.47	0.19**	0.010	0.17**	0.034	1,249
		(−0.12 to 0.25)		(0.05 to 0.33)		(0.01 to 0.33)		
	SDQ Total Difficulties^a^^,^^b^	0.02	0.84	0.07	0.46	0.02	0.82	1,249
		(−0.16 to 0.20)		(−0.12 to 0.26)		(−0.16 to 0.21)		

The 95% CIs (in parentheses) and *p*-values are adjusted for clustering at the town level. All scores are standardised non-parametrically with respect to age and have 0 mean and unit variance in the control group. Estimates control for baseline levels of cognitive, receptive language, expressive language, fine motor, and gross motor development, as assessed by the Bayley-III; the difficult, unadaptable, unstoppable, and unsociable scales in the Infant Characteristics Questionnaire; children’s sex; and tester dummies. Scoring of all measures outlined in S1 Table.

**p < 0.05: 2-tailed p-values for difference compared to control group.

^a^Measure scored such that higher values indicate more problems/lower levels of behavioural development.

^b^Total Difficulties subscale aggregates 4 of the SDQ subscales: Hyperactivity, Emotional Symptoms, Conduct Problems, and Peer Problems, and therefore is not contained in the behavioural factor.

Bayley-III, Bayley Scales of Infant and Toddler Development–Third Edition; CBQ, Children’s Behavior Questionnaire; SDQ, Strengths and Difficulties Questionnaire.

We note that *p-*values for testing the null hypotheses that the interventions had no impact on the 2 child development factors after 2 years would be even larger if we accounted for the fact that we tested multiple hypotheses (3 treatments and 2 outcomes).

There was no impact of either intervention on the FCI subscales Variety of Play Materials and Variety of Play Activities or on maternal depressive symptoms (Table 4).

**Table 4:** Estimated treatment effects for stimulation in the home environment and maternal depressive symptoms.

Outcome	Test/subscale	Stimulation	*p-*Value	Supplementation	*p-*Value	Stimulation and supplementation	*p-*Value	*n*
**Home stimulation**	FCI Variety of Play Activities	−0.00	0.99	0.16	0.19	0.16	0.16	1,249
		(−0.25 to 0.24)		(−0.08 to 0.40)		(−0.06 to 0.38)		
	FCI Variety of Play Materials	−0.11	0.36	0.05	0.73	0.17	0.19	1,249
		(−0.36 to 0.13)		(−0.23 to 0.32)		(−0.09 to 0.43)		
**Maternal depressive symptoms**	CES-D 10	0.06	0.50	0.06	0.52	0.02	0.88	1,213
		(−0.12 to 0.24)		(−0.13 to 0.26)		(−0.18 to 0.21)		

The 95% CIs (in parentheses) and *p*-values are adjusted for clustering at the town level. FCI scores are standardised non-parametrically with respect to age, and all scores have 0 mean and unit variance in the control group. FCI estimates control for baseline levels of the same 2 subscales (Variety of Play Materials and Variety of Play Activities), children’s sex, and tester dummies. CES-D 10 estimates control for baseline levels of the same scale. Scoring of all measures is outlined in S1 Table.

CES-D 10, Center for Epidemiologic Studies Short Depression Scale; FCI, family care indicator.

## Discussion

We assessed the medium-term effects of a scalable home-visiting intervention aimed at promoting Colombian children’s development through increasing the PS they experienced in their home environment. The intervention was based on a model that has been shown to have long-lasting impacts when implemented on a small scale and in a tightly controlled manner. This is the first randomised and sufficiently powered study we know of to assess whether such models can deliver sustained benefits when delivered at a larger scale through the local institutional infrastructure of existing government services. Two years after the intervention ended, we found no effects on children’s cognition, language, school readiness, executive functioning, or behavioural development. The lack of impacts is disappointing and occurred in spite of an improvement in cognition, receptive language, and the quality of the home environment at endline (end of intervention delivery) [19,23]. This study was based on a cluster randomised controlled trial with a large sample size, the measures appeared valid, and dropout was small and balanced, suggesting that our findings are internally valid. Likewise, take-up of the intervention in the treatment arms was near universal, and the intensity of exposure was high. In this discussion we therefore focus on how challenges in maintaining fidelity to evidence-based models when operating in a scalable fashion may have contributed to a lack of effects after 2 years and how such challenges might be overcome.

These results contrast with the 4 studies that we identified in our review of the literature, which found positive medium- or longer-term impacts of PS on child development [11–14,17]. In particular, they contrast with 2 randomised controlled trials in Jamaica that evaluated similar interventions—one trial, with low birth weight children born at term, found medium-sized impacts on child cognition and behaviour at age 6 years [13]; the other trial, with stunted children, followed participants to 22 years of age and found significant benefits to IQ, depression, educational attainment, behaviour, and wages [11,12]. A plausible interpretation of our results is that the short-term improvements to children’s development seen in our initial study were too small to be sustained 2 years later. The stimulation intervention had short-term benefits to cognition of 0.26 SD and to receptive language development of 0.22 SD [19]. While significant, these short-term effects were substantially smaller than the effects seen in the 2 Jamaican studies (0.91 SD [21] and 0.42 SD [44]), the Pakistan study (0.6 SD [18]), and an earlier Colombian study (0.90 SD [45]). Efficacy trials in the US that had long-term benefits also had large initial cognitive effects, whereas the impact of programmes that had small initial effects faded over time [46]. Nevertheless, the short-term effect sizes in the present study were similar to the weighted mean of 10 short-term effect sizes from home-visiting stimulation interventions calculated in a recent meta-analysis (0.32 SD) [3]. It is therefore unknown whether relatively small initial effect sizes lead to sustained improvements, and there is an urgent need for more follow-up studies before we can extrapolate from small short-term effects to sustained benefits.

This was one of the first attempts to implement the Jamaican curriculum approaching scale, and several lessons were learnt that should help improve the size of the child benefits in future scale-ups. We lacked resources to pilot the programme in Colombia, but beginning in a smaller area where implementation problems could be solved before expanding would have been desirable.

When going to scale with an evidence-based intervention, outcomes are affected by the fidelity of the implemented programme to the main components of the evidence-based model [47]. Fidelity is usually attained by providing coaching, training, and technical assistance to the front-line staff and supervisors [47] and by monitoring progress with continued on-the-job feedback. Staff turnover proved to be a substantial challenge in going to scale, and plans need to be in place to manage it. Unfortunately, FeA lacked capacity in ECD and could only assist in identifying participants and Madres Líderes but not supervisors, who we therefore hired from Bogotá. The intervention covered a vast area, and supervisors had to travel large distances to meet the Madres Líderes. Therefore, the frequency of contacts was planned for once every 6 weeks, compared to weekly in Jamaica, but in practice averaged once every 9 weeks. After the initial 6-week training, supervisors themselves had little support, apart from 2 refresher workshops. The only criterion for selection of Madres Líderes was their ability to read. Although Reach Up was designed for use with home visitors with low educational levels, it was intended for them to have frequent coaching and support from supervisors, with ideally weekly contacts. Both the visitors and supervisors probably needed more support. One solution would have been to select local supervisors (if necessary with lower qualifications), in order to limit travel and increase the frequency of supervision, thus improving the skills, motivation, and feelings of relatedness of the Madres Líderes. Furthermore, there were 3 Madres Líderes per town, and all were employed part time, each visiting 4 to 5 families. It is likely that full-time visitors would have had more commitment to the programme, and it would have been more manageable for supervisors to supervise fewer full-time visitors. Where possible, it may also help to increase the home visitors’ required level of skills for employment.

While our intervention operated through the institutional infrastructure of an existing government programme, it was time-limited and served only 15 FeA beneficiaries per town. It is possible that some of the implementation challenges would be reduced if the intervention became government policy. For example, if all beneficiary children of FeA of eligible age were offered the programme, then supervisors’ work would be based entirely within a single town, facilitating more frequent contact. Moreover, if the intervention were permanent and offered longer-term employment, it might reduce staff turnover. Likewise, as home visitors and supervisors gained experience, the quality of the intervention may have increased with time. Nevertheless, new challenges would surely emerge. There is limited information on implementation of early childhood stimulation programmes at scale in LMICs, and priorities for research are identifying how to maintain intervention fidelity and attain good and sustainable child benefits.

Other possible reasons for small effects are that child characteristics may affect outcomes, and evidence suggests that the more disadvantaged children benefit the most [7,48]. Whereas the Jamaican studies often targeted undernourished children with low levels of cognitive development, this study’s population was less disadvantaged. Duration of the intervention may also be related to sustainability of effects [7,14], and the Colombian intervention was slightly shorter than those in the Jamaican studies. Sustainability may also be affected by subsequent schooling and life experiences. Although preschool attendance was high in our sample (74.9%), it was not as high as in Jamaica, where all but 1 child attended preschool, which may have helped sustainability.

In our literature review, only 1 trial, similar to this study, integrated an intervention that could plausibly be delivered at scale into a government programme. The study, located in rural Pakistan, found evidence that monthly home visits and group sessions by health workers that promoted PS improved child development 2 years after the end of the intervention [17]. Health workers are familiar with working with families and children, and it may be easier to integrate into services with this experience rather than into ones with no such experience. However, the Pakistani study was de facto non-experimental in that there were 4 regions, each of which was allocated as a group to one of three treatment arms or the control arm [18].

Our curriculum was designed to improve parenting practices, and at endline we found it increased the variety of children’s play materials and play activities. However, effects had disappeared 2 years after the intervention ended. The lack of sustained impact on home stimulation implies that many children would have graduated from the intervention into unstimulating environments, where their development would not be supported. We need research on ways of improving parental involvement after the intervention. It may be necessary to provide ongoing intervention through to school entry.

Finally, it is possible that intervention effects may appear at a later age. Cognitive benefits from early childhood interventions usually decline over time [14,46]; however, sleeper effects in other outcomes may emerge later [46]. For example, in the Perry Preschool programme, in the US, effects on cognition faded and were no longer significant after the short-term evaluation, but large benefits were seen in terms of education, earnings, and social outcomes up to age 40 years [49]. In the Jamaican study on stunted children, impacts on IQ were smallest when the children were around 7 years old and not significant. However, other benefits appeared, and the IQ effects increased in subsequent follow-ups [1]. This creates a compelling argument for continuing to follow these children.

This study provides robust evidence on the medium-term effects of a PS intervention integrated into a government service delivered across 48 towns in a middle-income country. Strengths of the study are the scalable nature of the intervention, the randomised design, the large sample size, the relatively low attrition rate, and the wide range of valid measures of children’s cognition, language, and school readiness. The study’s limitations include that measures of behavioural development were collected by maternal report, which, although few alternatives exist for young children in large-scale studies, may not be as valid as direct observations [50]. This may have limited our ability to detect intervention effects on social, emotional, behavioural development, and self-regulation, developmental domains that are increasingly being considered as key mechanisms for how early environments affect later life outcomes [50–52]. Additionally, even with the large sample size, very small improvements in cognition, language, and executive function may have been missed due to power. A further limitation is that although we suggest that a plausible cause of the lack of sustained benefits from this intervention might be a lack of fidelity to core principles of the evidence-based model on which it was based, we lack comparable quantitative indicators of the quality of home visits across studies that could be used to assess this hypothesis.

Available evidence suggests that the design and implementation of larger-scale programmes present many challenges, and there is no guarantee that interventions that are effective in small efficacy trials will continue to be so when taken to scale. The lack of a sustained effect in this study, at least at 2 years post-intervention, is a warning against rushing to take interventions to scale before careful implementation research, which must consider all aspects of intervention delivery, especially providing rigorous supervision, coaching, and support for the home visitors and supervisors and linking with an institution that has an interest and skills in early childhood. There may also be scope for complementary interventions at later ages.

## Supporting information

S1 Alternative Language Abstract Spanish translation of the abstract by Gabriela Smarrelli. (PDF)

S1 Appendix Scripts for tracking telephone call and text messages. (PDF)

S2 Appendix Report on piloting activities. (PDF)

S3 Appendix Protocol for initial study and short-term evaluation. (PDF)

S4 Appendix ‘Scope of work’ from funding proposal. (PDF)

S5 Appendix Construction of scores using IRT and GRM. (PDF)

S6 Appendix Validation of child development measures (contains Tables A, B, and C). (PDF)

S7 Appendix Comparison of short-term and medium-term effect sizes (contains Table D). (PDF)

S1 Table Measures of primary and secondary outcomes. (PDF)

S2 Table Differential attrition by treatment status and baseline characteristics. (PDF)

S3 Table Exploratory factor analysis of all measures of child development. (PDF)

S4 Table Child development by maternal education. (PDF)

S1 Data Analysis dataset. (ZIP)

## Abbreviations

Bayley-III: Bayley Scales of Infant and Toddler Development–Third Edition
CBQ: Children’s Behavior Questionnaire
CES-D 10: Center for Epidemiologic Studies Short Depression Scale
Daberon-2: Daberon Screening for School Readiness–Second Edition
ECD: early childhood development
FCI: family care indicator
FeA: Familias en Acción
GRM: graded response model
IRT: item response theory
LMICs: low- and middle-income countries
MN: micronutrient
PS: psychosocial stimulation
PTT: pencil tapping task
SDQ: Strengths and Difficulties Questionnaire
WM: Woodcock–Muñoz
TVIP: Test de Vocabulario en Imágenes Peabody
