# Supplementary material for: Impacts 2 years after a scalable early childhood development intervention to increase psychosocial stimulation in the home: A follow-up of a cluster randomised controlled trial in Colombia
Source: PLoS Med. 2018 Apr 24;15(4):e1002556. doi: 10.1371/journal.pmed.1002556 (PMC5915272; doi:10.1371/journal.pmed.1002556)
Supplement: S1 Alternative Language Abstract — (PDF) [file pmed.1002556.s001.pdf]

Abstract

Antecedentes

Los bajos niveles de desarrollo infantil temprano (DIT) observados en países de ingresos bajos y medios es un tema de gran preocupación. Por ello, existe interés por universalizar el acceso a intervenciones de DIT, integrándolas a los servicios que ya ofrecen los gobiernos. Sin embargo, la evidencia de los efectos de mediano y largo plazo de modelos de intervención escalables es escasa. En un estudio anterior, mostramos los impactos positivos de una intervención que ofrecía actividades de estimulación psicosocial (ES) integrada a un programa de transferencias condicionadas en Colombia, en el desarrollo cognitivo y el lenguaje receptivo de los niños, así como en las prácticas de estimulación temprana en el hogar. En el presente estudio, analizamos los efectos de mediano plazo (dos años después) de la intervención en el desarrollo cognitivo, el lenguaje, el aprestamiento escolar, la función ejecutiva y el comportamiento del niño.

Metodología y Resultados

Los participantes del estudio fueron 1.419 niños de entre 12 y 24 meses de edad cuando se llevó a cabo la línea de base, que vivían en hogares beneficiarios del programa de transferencias condicionadas en 96 municipios de Colombia. Entre el 2009-2011, los 96 municipios (conglomerados) fueron asignados aleatoriamente a uno de cuatro grupos posibles: control (N=24, n=349); ES (N=24, n=357), suplementación nutricional de micronutrientes múltiples (MN) (N=24, n=354); y ES y MN combinados (N=24, n=359) (ensayo de control aleatorio por conglomerados). Las intervenciones duraron 18 meses. En el presente estudio, dos años después del fin de las intervenciones (26 de septiembre del 2013 a 11 de enero del 2014), evaluamos los impactos en el desarrollo cognitivo, de lenguaje, el aprestamiento escolar, la función ejecutiva y el comportamiento cuando los niños tenían entre 4.5 y 5.5 años. Los evaluadores encargados de administrar las pruebas desconocían la asignación de los grupos al tratamiento, pero no así los participantes. Se evaluó al 88.5% de los niños en el estudio original (n=1256) y los datos se analizaron en base a la *intención al tratamiento* (*intention-to-treat*). Los puntajes de las pruebas se examinaron utilizando análisis factorial y se obtuvieron dos factores: cognitivo (cognición, lenguaje, aprestamiento escolar y función ejecutiva) y de comportamiento. Dos años después de la intervención, no encontramos impactos

de las intervenciones en el factor cognitivo (ES: -0.031 DE, 95% IC -0.229-0.167; MN: -0.042 DE, 95% IC -0.249-0.164; ES y MN: -0.111 DE, 95% IC -0.311-0.089), ni en el factor comportamiento (ES: 0.013 DE, -0.172-0.198; MN: 0.071 DE, -0.115-0.258; ES y MN: 0.062 DE, -0.115-0.239), ni tampoco en las prácticas de estimulación temprana en el hogar. Las limitaciones del estudio incluyen que la medición del comportamiento del niño se obtuvo por reporte materno y que los efectos de menor magnitud pueden no haber sido captados, a pesar del tamaño de muestra.

Conclusiones

No hallamos evidencia de que los impactos de la intervención de ES se sostuvieran dos años después que ésta finalizara. Esto puede deberse a que los efectos iniciales de la intervención fueran demasiado pequeños para mantenerse en el tiempo, o a la falta de continuidad en las prácticas de estimulación temprana en los hogares. Ambos factores están probablemente relacionados con las concesiones que uno debe hacer cuando se implementan intervenciones de DIT a mayor escala y de forma escalable. Ello sugiere que no se debería extrapolar los efectos de mediano plazo obtenidos en ensayos de eficacia a pequeña escala (*efficacy trials*) a los que pueden generar intervenciones de mayor escala implementadas de forma escalable. A futuro, es importante analizar en mayor profundidad las diferencias que existen entre uno y otro tipo de intervenciones para el diseño e implementación de programas y políticas de DIT efectivas.

Trial registration: ISRCTN18991160.
