# Supplementary material for: Impacts 2 years after a scalable early childhood development intervention to increase psychosocial stimulation in the home: A follow-up of a cluster randomised controlled trial in Colombia
Source: PLoS Med. 2018 Apr 24;15(4):e1002556. doi: 10.1371/journal.pmed.1002556 (PMC5915272; doi:10.1371/journal.pmed.1002556)
Supplement: S1 Appendix — (PDF) [file pmed.1002556.s002.pdf]

S1 Appendix: Scripts for Tracking Telephone Call and Text Messages

GUIÓN DE LLAMADA TELEFÓNICA – PROYECTO PILOTO ESTIMULACIÓN TEMPRANA ARD-334-3

Muy buenos días o tardes, por favor me puede comunicar con la Señora ________ (*madre del niño o quien respondió la encuesta en LB*).

SI NO ES LA PERSONA, ENTONCES INDAGUE EN QUÉ MOMENTO LA PUEDE CONTACTAR, ACTUALICE O SOLICITE NÚMERO TELEFÓNICO, DÉ LAS GRACIAS Y TERMINE:

SI QUIÉN RESPONDE ES LA SEÑORA O CUIDADOR DEL NIÑO(A), LEA EL SIGUIENTE TEXTO,

Mi nombre es __________, la estoy llamando de Sistemas Especializados de Información SEI s.a., la empresa que los visitó a comienzos del año 2010 y que luego volvió en entre septiembre y diciembre de 2011 para el Estudio de Desarrollo Infantil Temprano que se adelantó junto con el IFS de Londres y el programa Familias en Acción del DPS.

Durante estas visitas se les tomaron medidas de peso y talla a usted y a (nombre del niño) y se les tomó una muestra de sangre. Luego llevaron al niño(a) a un sitio en donde una psicóloga jugó con ellos y le hizo a usted algunas recomendaciones ¿si lo recuerda?

SI LA PERSONA DICE QUE SÍ RECUERDA, ENTONCES LEA:

El motivo de mi llamada es actualizar los datos de ubicación de su hogar (dirección, teléfonos fijos o celulares o municipio de residencia) pues vamos a visitarlos nuevamente, entre septiembre y diciembre de este año y quisiéramos estar seguros de que vamos a poder encontrarlos.

ACTUALICE LA INFORMACIÓN EN LA TABLA "Actualizacion_Informacion_del_hogar_LB_PS"

Los datos se verifican uno a uno según tabla "Actualizacion_Informacion_del_hogar_LB_PS.xls"; Nombres y apellidos, lugar de residencia, teléfonos fijos y celulares, correo electrónico y fecha de nacimiento de la madre y del niño de seguimiento. Se les pregunta si hay otro número de celular o teléfono fijo para registrarlo.

Con respecto a los contactos, se le mencionan los nombres de las personas que aparecen en la tabla y se les indaga si se dejan esos o se cambian por unos nuevos. Si cambian estos contactos se les pide que nos den los nombres, números telefónicos fijos y celulares, direcciones de tres personas que nos puedan dar su dirección y número telefónico en caso de que el hogar cambie de residencia. Si no cambian los contactos, solicitamos la información de una tercera persona para completar los tres.

Se indaga si el niño aún vive en el hogar, de no ser así, se solicita información de los datos para visitar el hogar donde se encuentra el niño/a actualmente.

SI NO ES POSIBLE ESTABLECER CONTACTO DIRECTO CON EL HOGAR O CON LAS PERSONAS DE CONTACTO, SE DEJA EL MENSAJE Y LOS NÚMEROS DE TELÉFONO Y NOMBRES DE LAS PERSONAS DE CONTACTO EN SEI.

UNA VEZ ACTUALIZADOS LOS DATOS LEA:

Además, quisiéramos pedirles que por favor, nos informen de cualquier cambio de dirección teléfonos o municipio de residencia, pues así nos facilitaran mucho la localización del hogar y garantizar que pueda participar en la próxima encuesta.

Para agradecer su compromiso con el estudio y que nos llame a comunicarnos cualquier cambio en sus datos de contacto, no sólo le reembolsaremos el costo de la llamada, sino que además le obsequiaremos con $5,000 co de recarga a su celular.

También como agradecimiento por su compromiso, haremos un sorteo de $35.000,oo entre un grupo de 50 mamitas que nos contesten esta llamada.

DESPUÉS DE LA VERIFICACIÓN DE LOS DATOS, LEA:

Señora _______ muchas gracias por atender mi llamada. Recuerde que vamos a estar visitando su hogar nuevamente entre septiembre y diciembre de este año.

RECORDARLE que por favor, nos mantengan informados acerca de si sus datos de contacto cambian – por ejemplo, si cambian de número de celular, de vivienda, si migran a algún otro municipio, etc. Recuerde que como muestra de nuestro agradecimiento por su compromiso con el estudio, NO SÓLO LE REEMBOLSAREMOS EL COSTO DE LA LLAMADA para notificarnos de un cambio en sus datos de contacto, SINO QUE ADEMÁS LE OBSEQUIAREMOS CON $5,000 COP DE RECARGA A SU CELULAR

English Translation:

SCRIPT OF TELEPHONE CALL– PROYECTO PILOTO ESTIMULACIÓN TEMPRANA ARD-334-3

Good Morning (or Good Afternoon), can you please put me in contact with Miss ________ (*carer of the child or the person that responded the baseline questionnaire)?*

IF THE PERSON IS NOT AVAILABLE OR COULD NOT BE CONTACTED, ASK WHEN WILL THE PERSON BE AVAILABLE. UPDATE OR ASK FOR A NEW TELEPHONE NUMBER. GIVE THANKS, AND FINISH THE CALL.

IF THE MOTHER OR CARER OF THE CHILD IS AVAILABLE AND ANSWERS THE CALL, READ THE FOLLOWING:

My name is __________. I am calling you from "*Sistemas Especializados de Información SEI s.a.*", the company that visit your house for the Study on Early Childhood Development developed by IFS and the programme Familias en Acción of DPS. We visited you at the beginning of 2010 and between September and deceiver of 2011.

During these visits, we took weight and height measures, as well as blood samples, of you and (name of the child). Afterwards, the child was taken to a different place

and a psychologist played with him/her. Then, we gave you some recommendations. Do you remember this?

IF THE PERSON SAYS YES, READ THE FOLLOWING:

I am calling you to update the information about the location of your house (address, phone, cell phones, and municipality of residence). We need this information because we will visit you again between September and December of this year.

UPDATE THE INFORMATION IN THE TABLE "Actualizacion_Informacion_del_hogar_LB_PS (Update_Information_of_household_LB_PS"

*All the data should be reviewed according to the table "Actualizacion_Informacion_del_hogar_LB_PS.xls (***Update_Information_of_household_LB_PS.xls***"; Names and Surnames, place of residence, phones and cell phones, emails and date of birth of the mother and the child. You should also ask about any additional phone numbers and include them in the tracking data.*

Concerning contact persons, you have to mention the names of the persons that appear in the table and ask if they wish to keep or change them. If they wish to change the contact persons, ask for the name, phone and the address of the 3 new contact persons that will be able to share with us the carer/mother address and phones. If they wish to keep the same contact persons, ask for the name, phone and address of a third contact person.

Ask if the child still lives in the household. If he/she is no longer living in the household, ask for the new location and update the information to be able to visit this household in the future.

IF IT IS NOT POSSIBLE TO CONTACT ANYONE IN THE HOUSE OR ANY OF THE CONTACT PERSONS, LEAVE A MESSAGE AND PROVIDE THE NAMES AND TELEPHONE NUMBERS OF THE CONTACT PERSONS AT SEI.

AFTER UPDATING THE INFORMATION, READ THE FOLLOWING:

We will be very grateful if you could please let us know in case you change your address, telephone or municipality of residence. This will help us to locate you and will ensure that you can participate in future surveys.

As a way of thanking you for your commitment to the study and for calling us in case you update your contact information, we will refund the costs of the call and we will give you $5,000 co to top up the credit of your phone.

Also, we will raffle $35.000,oo among 50 mothers/carers that answer this call.

AFTER VERIFYING THE INFORMATION, READ THE FOLLOWING:

Miss _______, thank you very much for answering this call. Please, remember that we will be visiting your house again between September and December of this year.

REMIND THEM to please let us know if they change their contact information. For instance, their phone, address, municipality of residence, among others. Remind them that to show our appreciation for their commitment to the study, and if they call us to update their contact information, we will NOT ONLY REFUND THE COSTS OF THE CALL, BUT WE WILL ALSO GIVE THEM $5,000 co TO TOP UP THE CREDIT OF THEIR PHONE.

Box A: Script for Tracking Telephone Call to Mother

CONTENIDO DE MENSAJE DE TEXTO ENVIADO EN LA FECHA DE CUMPLEAÑOS DEL NIÑO:

Hola XXX hoy estas de cumpleaños! Que pases muy feliz con toda tu familia. Un abrazo del IFS-Acción Social y SEI sa. Si cambian de casa avisarnos al XXX.

English Translation:

TEXT MESSAGE SENT THE DAY OF THE BIRTHDAY OF THE CHILD:

Hi XXX, today is your birthday! We hope you have a beautiful day with your family. Hugs from IFS-Acción Social and SEI sa. If you moved, please let us know at XXX.

Box B: Script for Text Message Sent on Child's Birthday

CONTENIDO DE MENSAJE DE TEXTO A ENVIAR EN EL DÍA DE LA MADRE A LA MAMÁ DEL NIÑO O SU RESPONSABLE DE CUIDADO:

Señora XXX, en esta fecha tan especial para las madres, el IFS-ACCIÓN SOCIAL Y SEI s.a. quieren expresarle a usted sinceras felicitaciones, y reconocer la gran labor de formar a sus hijos con buenos principios y valores, herramientas indispensables para tener ciudadanos de bien en el futuro.

Reciba un fuerte abrazo.

English Translation:

TEXT MESSAGE SENT ON MOTHERS DAY TO THE MOTHER OF CARER OF THE CHILD:

Miss XXX, on this special day, IFS-ACCIÓN SOCIAL and SEI s.a. want to congratulate you for the amazing work that you do to raise your children, for teaching them good values and providing them with the tools they need to become good citizens.

Kind regards.

Box C: Script for Text Message Sent on Mother's Day
