# Supplementary material for: Impacts 2 years after a scalable early childhood development intervention to increase psychosocial stimulation in the home: A follow-up of a cluster randomised controlled trial in Colombia
Source: PLoS Med. 2018 Apr 24;15(4):e1002556. doi: 10.1371/journal.pmed.1002556 (PMC5915272; doi:10.1371/journal.pmed.1002556)
Supplement: S2 Appendix — (PDF) [file pmed.1002556.s003.pdf]

Report on piloting activities – January 2015

Does a home visiting programme in early childhood have sustained effects on development 2 years after it ends? Evidence from Colombia.

Piloting of the instruments used in the second follow-up took place in poor neighbourhoods in Bogota and the nearby town of Sesquilé during August and September 2013. We piloted psychometric assessments on child and mother, collection of DNA material via saliva swaps, beliefs data, and some of the more standard household and community socio-economic modules.

Piloting of Psychometric Assessments

Piloting of those psychometric assessments that required direct administration on the child was conducted by [redacted] and [redacted], both psychologists and consultants to the project. Marta Rubio-Codina (co-I) piloted those psychometric tests administered by maternal report. [redacted] (the psychologist that conducted the testers' trainings) and Marta coordinated the piloting exercise and made decisions on modifications. Sally Grantham-McGregor (co-I) provided overall supervision from London.

The purpose of the piloting was to determine what tests to include, and which items within, as part of the second follow-up assessments. Criteria taken into consideration to decide what tests to keep included: (i) adequacy of the tests' difficulty level for children 4.5 to 5.5 years old living in similar socio-economic environments as the target child; (ii) enjoyment of the child during the test; and (iii) test length, so as to ensure a total testing time with the child of at most 1.5 hours. The pilot was also extremely useful to determine the appropriateness of the language used and identify suitable wording in Colombian Spanish, finalise the list of test materials required, and identify the best order for administration of the tests.

Piloting activities were carried out in 2 child care centres and 2 Hogares Comunitarios in poor neighbourhoods in Bogota. Maternal/main caregiver consent to participate in the piloting was obtained, either directly or through the director of the child care centre.

Tests on target child by direct assessment

[redacted] and [redacted] piloted the following scales and subscales on a sample of n = 20-30 children, depending on the test.

- *Woodcock-Munoz – Cognitive Battery (including Supplement)*

  The following items were piloted and kept:
  - Item 5: fluid reasoning/fluid intelligence, as it offered good variability and had been used in Colombia before.
  - Item 6: visual perception/processing speed, as children enjoyed it and had been used in Colombia before. We reduced testing time to 1 min to make it slightly harder.
  - Item 12: fluid reasoning, use 'food and drinks', 'animals' and 'clothes' as categories; and 'body parts' as the example item.
  - Item 13: visual perception/spatial memory, offers nice variation/discriminatory ability.

- Item 16: visual perception/processing speed. Children enjoyed matching items; plus, it was felt it implied a change from previous activities. It was decided children should strike pictures as opposed to circling them (which was much harder for the child and took a lot of time).
- Item 21 and 21b: long-term retrieval and longer term memory (learning) – despite it being long the children found it very engaging and enjoyable.

The following items were piloted and dropped because they were too difficult:

- Item 11: knowledge comprehension. Also, it was considered the Daberon-II already included some knowledge questions that could discriminate well amongst children.
- Item 17: short memory test (words).
- Item 18: processing speed/naming pictures.
- Item 20: attention.
- Item 22: visual perception.

- *Woodcock-Munoz – Achievement Battery*

The following items were piloted and included:

- Item 10: quantitative skills. We have removed some items already covered in Daberon-II but added 3 items on counting and number recognition as data showed that they may help identify very bright children (see more details below).
- Item 14: expressive language

The following items were piloted and dropped:

- Item 15: comprehension and knowledge – children did not understand what they were supposed to do. Also, it was considered the Daberon-II already included some knowledge questions that could discriminate well amongst children.
- Item 18: quantitative skills. Very few items offered enough variability; those that did were already covered in the Daberon-II (see below).

- *School Readiness*. Piloting the *Daberon-II* confirmed it is an easy test to give, that offered enough variability, which seemed to add information with respect to other tests. We removed very easy and very difficult items that showed no variability, to reduce testing time while maximising test information content. We left a few easy items to make sure all children (even the slower ones) enjoyed the test. We also left a few of the more difficult items – which very few children could do – to make sure we identified the very bright children. We also made some minor modifications to the language in some items to reflect the use of Colombian Spanish and the child's immediate environment.

- *Inhibitory Control.* We piloted both the Head Toes Knees and Shoulder (HTKS) test and the Pencil Tapping Task (PTT). We kept the PTT since it offered more variability for children of these ages. HTKS seemed too hard for the median child in the pilot sample.

The following table includes the final list of tests and subtests we decided to include, including administration order, times, and comments on modifications. Note that we did not pilot *the Test de*

Vocabulario de Imágenes de Peabody, which we used to assess receptive language levels since this is a standardised test that has been widely used in Colombia for children of these ages before.

TEST	Time		Comments
**Presentation** (Chat with Mum) & Let Child Scribble (or blocks to play with)	4		
**Daberon II (adapted)**	18	**30**	simplified to 83 items + 3 items from WM ACH 10
**WM ACH 14** -- expressive language	3		
**PTT** (Pencil Tapping Task)	2		
**WM COG 13** -- visual spatial perception/spatial memory	5		
**WM COG 12** -- fluency, ability to classify, recall what you know and process it quickly	2		food and drinks, animals, clothes (and use body parts as an example)
(break: juice + cookie)	*5*		
**WM COG 5** -- fluid reasoning/non-verbal reasoning/executive processing ('same and different' shapes/rule why different)	3	**34**	
**WM COG 6** -- visual perception/visual processing speed -- 'point at same shapes'	1.5		allow 1 min
**WM COG 16** -- processing speed	2.5		*allow 2 min*
**WM COG 21** -- long term retrieval (martians)	10		
(mini break + stretch) flexible depending on child's needs (avoid if possible)	2		
**Peabody**	14		
**WM COG 21b** -- long term memory (3 marcianos)	1		ask about 3 martians that appear before the first celing - is this standardised in the manual in any way?
**TOTAL TIME**	**73**		
**Wolke (modified)** (tester ratings)	**3**		

Tests on target child by maternal report, and tests on mother

Marta Rubio-Codina administered the following assessments on 20 to 30 mothers, depending on the test (some tests required less administrations as decisions on modifications/requirements were more obvious):

- Children Behaviour Questionnaire (CBQ), child attention and inhibitory control subscales by maternal report. Some modifications were made to the wording of some of the items.
- Strengths and Difficulties SDQ (SDQ) on child behaviour, by maternal report, to ensure comprehension.
- Ravens Standard Matrices to asses maternal IQ, by direct assessment on the mother. Given time constraints, we decided to keep the 3 middle series (out of a total of 5), consisting of 12 matrices each. The easiest and hardest series of matrices did not show much variability. We also piloted the Coloured Matrices but the series of matrices therein were found to be too easy.
- Big Five Inventory (BFI) to measure maternal personality. It was found that mums really enjoyed this test. We modified certain words to better reflect Colombian Spanish and facilitate comprehension.
- Maternal Knowledge on Child Development. We made some minor modifications to the wording. We had used this tool at baseline and first follow-up but were still concerned at maternal comprehension of the items.
- CESD-10 to measure maternal depressive symptoms. While we have used this measure intensively in this sample, we wanted to check the wording of a couple of items.

We did not pilot measures on the quality of the home environment (level of stimulation in the home), since we had already used these questions at baseline and first follow-up and they had worked well. Moreover, it would have required going to the homes, which was not possible given time constraints.

Tests on sibling by maternal report

We had proposed to assess child development on the target child's immediate younger sibling using the third edition of the Spanish Ages and Stages Questionnaire (ASQ-3) and his/her level of vocabulary using the MacArthur-Bates Communicative Development Inventories (CDIs I, II and III). Both tests had been previously used by members of the research team in Colombia on children of these ages and similar socio-economic status. Hence, no piloting was deemed necessary.

Piloting of Other Modules: Beliefs, DNA material, and household/community SES modules

[redacted] (research assistant) and [redacted] (from the data collection company SEI) provided piloting supervision of the modules on beliefs, DNA material, and standard modules in the household survey. Orazio Attanasio (PI) provided overall supervision from London and on-site.

DNA material. This involved collection of buccal cell samples from the target children using saliva swabs. N=60 children aged 4-5.5 years were selected from 4 childcare centres (jardines infantiles) to participate, often after having participated in the piloting of the psychometric tests. They were accompanied by their mothers or main caregivers. Participants consented to participate in the piloting of DNA extraction, after the procedure had been thoroughly explained to them. Mothers were reminded of their right to withdraw from the piloting at any one stage.

Standard protocols for collection of this type of material were piloted. The piloting was useful to define the best way in which to transmit instructions to the mothers/main caregivers. In this way, it was decided that it would be best to have the mother first collect her own saliva sample (copying what the interviewer was doing), and then her child's, in order to avoid any direct contact between the interviewer and mother/child (unless the mother requested assistance). Moreover, and in order to ensure standardised conditions for everyone in the sample, it was decided that the saliva collection would be done in a community centre (as opposed to the child's home). Pictures with examples on the protocols to follow would be displayed on the centres' walls. Finally, it was observed that some mothers were reluctant to have their or their children's saliva samples taken. Hence, in order to minimise sample loss, it was decided that genetic material would be collected at the end of all other surveys and assessments, after signature of an independent consent form.

Maternal beliefs on child development. The second measure to be piloted was a new set of questions about mothers'/main caregivers' beliefs. Approximately 60 mothers/main caregivers with children aged 4-5.5 participated in the piloting. As before, participants were selected from childcare centres. A team of 3 interviewers hired by SEI supported Pamela Jervis and Fernando Trujillo in the piloting of this module.

The objective of this module is to assess maternal beliefs regarding the development of skills in early childhood and expectations on the returns to investments (i.e. the importancet of material investments, spending quality time with the child, and going to preschools of different perceived quality).

The pilot was extremely useful to define the final manipulative (tool) to use during the assessment. This consisted of a wooden board with horizontal strings and one bead for each question. On the top

there were the ages of the children – which reflected the range of possible answers to the question. Mothers/main caregivers move the bead along the string for each question to reflect her answer and, more importantly, modify them as her answers are updated/modified as part of the natural process of the assessment.

There were five additional lessons resulting from the piloting. First, it was deemed crucial to do some practice with the mother before the start of the assessment. Second, the need to use situations that mothers/main caregivers could easily understand (i.e. age children will start to crawl, walk and run) as examples during this practice. Third, it was observed that mothers/main caregivers could more easily respond in terms of month-of-age than range of months-of-age. Fourth, a need to show illustrations while each scenario/question was being asked/explained was identified. The set of illustrations that all mothers could relate to was also finalised during the pilots. Finally, it was deemed crucial to standardise the protocols and examples used by the interviewers to explain the concepts and support mothers in the assessment, as well as the way in which questions were asked.

Other household and community modules.

There were main two modifications to the community questionnaire with respect to the ones used at baseline and first follow-up:

1. Information on educational services provided in the community. Pamela Jervis defined the questions after visiting 3 towns where she enquired in child centres/institutions what information they could provide and the best form of asking about it regarding time in operation, number of children attending, range of ages, number of professionals in the institution, monthly fees, opening times, etc.
2. Questions on prices for learning materials such as toys, books and clothes for 5 year olds. A team of 2 interviewers employed by SEI visited 3 towns, similar to the ones in the sample, to find out the best way of asking for this information (whether to refer to specific toys and if so, which ones).

Regarding the household questionnaire, some questions were removed and some others were marginally edited, on the basis of the analysis of the information collected in previous rounds.
