# Supplementary material for: Impacts 2 years after a scalable early childhood development intervention to increase psychosocial stimulation in the home: A follow-up of a cluster randomised controlled trial in Colombia
Source: PLoS Med. 2018 Apr 24;15(4):e1002556. doi: 10.1371/journal.pmed.1002556 (PMC5915272; doi:10.1371/journal.pmed.1002556)
Supplement: S3 Appendix — (PDF) [file pmed.1002556.s004.pdf]

Spirit Checklist

(compiled from http://www.spirit-statement.org/title/)

Administrative Information

1: Title
Using a conditional cash transfer programme to scale up an integrated early child development intervention in Colombia: a cluster randomised controlled trial

2: Trial Registration
"Early childhood development: Identifying successful interventions and the mechanisms behind them", Controlled trials ISRCTN18991160.

World Health Organization Trial Registration Data Set	
**Primary Registry and Trial Identifying Number**	Trials ISRCTN18991160
**Date of Registration in Primary Registry**	18 December, 2009
**Secondary Identifying Numbers**	UCL Ethics 1827/001
**Source(s) of Monetary or Material Support**	Economic and Social Research Council
**Primary Sponsor**	Institute for Fiscal Studies
**Secondary Sponsor(s)**	Economic and Social Research Council
	Inter-American Development Bank
	International Growth Centre
	World Bank
**Contact for Public Queries**	Emla Fitzsimons
	emla_f@ifs.org.uk; [redacted]
	IFS, 7 Ridgmount St, London WC1E 7AE

**Contact for Scientific Queries**	**Principal Investigator** Emla Fitzsimons emla_f@ifs.org.uk; [redacted]; Institute for Fiscal Studies, 7 Ridgmount St, London WC1E 7AE
**Other scientific contacts**	Orazio Attanasio [redacted] Dept of Economics, University College London, Gower St, London WC1E6BT
	Marta Rubio-Codina marta_r@ifs.org.uk; [redacted]; Institute for Fiscal Studies, 7 Ridgmount St, London WC1E 7AE
**Public Title**	Using a conditional cash transfer programme to scale up an integrated early child development intervention in Colombia: a cluster randomised controlled trial
**Scientific Title**	Using a conditional cash transfer programme to scale up an integrated early child development intervention in Colombia: a cluster randomised controlled trial
**Countries of Recruitment**	Colombia
**Health Condition(s) or Problem(s) Studied**	Early child cognitive, language, and motor development; physical growth (height and weight for age); health (anemia)
	**Intervention(s)** Integrated early child development intervention
	Psychosocial stimulation,

	micronutrient supplementation, both interventions, no treatment
**Key Inclusion and Exclusion Criteria**	Ages eligible for study: 12-24 months<br>Sexes eligible for study: both<br>Inclusion criteria: child's mother a beneficiary of the conditional cash transfer programme Familias en Accion
**Study Type**	Interventional Allocation: randomized<br>Intervention model: factorial<br>Masking: none
**Primary purpose:**	Secondary prevention
**Date of First Enrollment**	1st February 2010
**Target Sample Size**	1,440
**Recruitment Status**	Complete: participants are no longer being recruited or enrolled.
**Primary Outcome(s)**	*Outcome Name:*<br>(1) Children's mental (cognitive), language and motor development<br>*Method of measurement:* Bayley Scales of Infant Development; MacArthur-Bates vocabulary check list.<br>Timepoint: 18 months after the start of treatment<br>(2) Children's nutritional status<br>*Method of measurement:* Haemoglobin: Hemocue method; Weight: scale (SECA 872); Height: wooden length boards (ShorrBoards), and

	children's consumption of iron rich food<br>*Timepoint:* 18 months after the start of treatment
**Key Secondary Outcomes**	Outcome Name: (1) Maternal depression<br>Method of measurement: the 10-item CESD Scale.<br>(2) Investigate the constraints that households face when making choices relevant to their children's development, and to investigate why the intervention works or not.

3: Protocol Version

Issue Date: 5th November 2013. Please note, this version puts together, in a coherent format, various protocol documents that were in place by January 2009 and that were followed throughout the course of this research.

Protocol Amendment Number: 00

Author(s): *O.A.; C.F.; E.F.; S.G; C.M.; M.R.C*
O.A Orazio Attanasio
C.F Camila Fernandez
E.F Emla Fitzsimons
S.G Sally Grantham McGregor
C.M Costas Meghir
M.R.C Marta Rubio-Codina

4: Funding

The study is funded by the Economic and Social Research Council (ESRC), the Inter-American Development Bank (IADB), the International Growth Centre (IGC), and the World Bank.

The ESRC contributes to intervention costs, evaluation costs, and research time.
The IADB, IGC and World Bank contribute to intervention costs.

5: Roles and Responsibilities

OA[*, **], CF[#], EF[*], SG[**], CM[+], MRC[*]

OA and EF conceived of the study. All authors initiated the study design and EF and MRC managed implementation. EF and OA are grant holders. CM provided statistical expertise in trial design and MRC conducted the primary statistical analysis. All authors contributed to refinement of the study protocol and approved the final manuscript.

*Institute for Fiscal Studies, London; **University College London; # Mathematica; +Yale University

Introduction

<u>6: Background and Rationale</u>

Introduction: It is well established that the first five years of life lay the basis for lifelong development. However, during this vital period many children in developing countries are exposed to poverty, malnutrition, illnesses, and un-stimulating home environments. These factors are likely to have a detrimental effect on children's cognitive, motor, and social-emotional development, as well as on their health, thus prohibiting them from reaching their full developmental potential (Grantham-McGregor et al. 2007). In particular, children growing up under these circumstances are likely to have poorer health, lower school achievement and lower earnings potential. As adults, they are less likely to provide adequate stimulation and resources for their children, thus contributing to the intergenerational transmission of poverty and economic inequality (Sen, 1999). This period of early childhood is therefore a critically important one for intervention (Nelson, 2000). Research provides evidence that interventions in this period are not only extremely important for brain and physiological development, but can also be feasible and relatively cost-effective (Heckman and Masterov, 2005).

Mechanisms: The most effective interventions provide direct learning experiences to children and their families (Engle et al. 2007). An example of such interventions is weekly home visits by well-trained professional personnel who interact with mothers and children (see Grantham-McGregor et al. 1991 on an intervention in Jamaica). Visits are intended to model and reinforce parenting behaviors that foster child development, and to promote emotionally-supportive and enriched maternal interactions with children. Moreover, many children are malnourished, which is likely to affect adversely their development.

Existing knowledge: There is robust evidence that well-targeted and well-designed interventions have positive impacts on the development of vulnerable children and some evidence that benefits are sustainable (Hoddinott et al. 2008). It has been amply documented also that nutritional

deficiency affects negatively and significantly cognitive as well as physical development (see Walker et al. 2007). There is also some evidence that improving nutrition has positive effects on children's health and physical and cognitive development, particularly for children under 3 (Martorell, 1995; Maluccio et al. 2006; Walker et al. 2007).

Need for a trial: Translating the existing evidence into effective policy requires information on how to scale up early childhood development (ECD) programmes that may reduce some of the developmental risks associated with poverty. A crucial challenge in scaling up programmes that have been shown to be successful is to achieve cost-effectiveness of ECD services whilst maintaining their quality and attaining similar impacts on child development to those identified in effectiveness studies. More evidence is needed to demonstrate that positive effects can be achieved when scaled-up ECD services are delivered under conditions that depart from those available in smaller well-controlled studies. Moreover, in terms of the interaction between nutritional status and stimulation, this could be positive either for biological reasons (iron deficient anaemic children are more tired and less active than non-deficient children (Lozoff et al. 1998) and maybe unable to fully utilise the intervention), or because mothers spend more time with the child, as they realise that their investment now has an extra return, due to the supplementation. There is a need to understand possible interactions or synergies between psychosocial stimulation and nutritional interventions.

Explanation for choice of comparators.

The control group represents the status quo, receiving neither home visits nor micronutrient supplementation.

7: Objectives

The key objective of the study is the identification of interventions that are effective at improving developmental outcomes of children, affordable because they use existing resources and networks, possibly in connection with an ongoing welfare programme, and implementable on a long term basis. This analysis can be extremely valuable and informative in a variety of different contexts where there exist good networks and strong ties amongst local people, as is the case in many developing communities. Specifically, the objectives are:

1. To design an integrated intervention to promote early childhood development (ECD) in conjunction with healthy nutritional status in rural Colombia. The psychosocial stimulation programme is based on the Jamaican

home visiting model, which demonstrated positive short- and long-term effects (Grantham-McGregor et al 1991; Walker et al 2006), but with important adaptations to the Colombian realities. The micronutrient supplementation consists of Sprinkles – encapsulated micronutrients in powder form (Zlotkin et al 2005) – developed to treat childhood anaemia.

We will exploit pre-existing resources and links in communities in rural Colombia in order to deliver these services in a cost-effective and sustainable way. The links we will exploit are due to the presence of a conditional cash transfer programme, Familias en Acción. Local mothers carry out the visits from amongst those attending the meetings organised in the context of this conditional cash transfer programme.

2. To measure the impacts of the intervention using a randomised controlled trial. We will measure their impacts on the development and nutritional status of children, including children's motor and mental development, language development and growth and haemoglobin status. We will also consider outcomes relating to mothers, such as maternal depressive symptoms.

3. To study why a particular intervention works or not. There are several channels through which the interventions can affect early childhood development, such as improved nutrition, increased awareness about the importance of stimuli, acquired knowledge on good stimulation practices, and so on. Our research, and in particular the experimental variation and the rich data we will collect, will allow us to construct and estimate models of individual investment behaviour that will help us distinguish among different hypotheses about the nature and origin of the effects we observe. The models will be crucial for the possibility of extrapolating what we learn on the specific interventions to different contexts.

<u>8: Trial Design</u>
Cluster randomised controlled trial, with a two-by-two factorial design with municipalities assigned to four groups; micronutrient supplementation, stimulation, combined interventions or control.

Methods: Participants, interventions, outcomes

<u>9: Study Setting</u>

The study is to take place in 96 municipalities in Colombia, located in three regions that are spread across eight of the country's 32 departments. Each municipality has a population ranging from 2,000 to 42,000 inhabitants.

10: Eligibility criteria

Patients (or a representative) must provide written, informed consent before any study procedures occur.

Inclusion Criteria

Individuals eligible for the trial must comply with all of the following **at randomisation**:

1. Age between 12 and 24 months
2. Mother is a beneficiary of the Familias en Accion conditional cash transfer programme.

Exclusion Criteria

To facilitate implementation of the programme, in particular given that it will be delivered by low-educated women with no prior experience in child development, individuals with the following characteristics will be excluded:

1. mental or physical disability as reported by the mother
2. living with another child who is less than 12 months apart in age (potential siblings and cousins)

11: Interventions

96 eligible clusters will be stratified by region (into 3 regions) and then randomised in equal proportions into stimulation, micronutrient supplementation, both combined, or control.

Within each cluster, a list of all madre lideres will be identified. Amongst the families she is responsible for, a list of all those including a child aged between 12 and 24 months will be identified. Five such households will be chosen at random, for each madre lider.

In stimulation and combined clusters, madre lideres will be asked to conduct weekly home visits to these families, for 18 months. If they cannot (due to time or literacy constraints), they will be asked to suggest a replacement. The madre lider or her replacement will visit all of the 5 families once per week, with each home visit lasting one hour. The content and structure of the home visits will follow the curriculum of Grantham McGregor for Jamaica.

In supplementation and combined clusters, the sprinkles will be delivered to homes by the madre lider (or her replacement) once per fortnight. Enough

sachets will be delivered to cover daily intake of the sprinkles for all children below 6 years of age. At the beginning of the study, home visitors (or replacements) will provide mothers with a monitoring booklet with use and storage instructions and forms for recording intake, which will be checked regularly. Note also that sachets will include a pictorial representation of use.

When a Madre Lider refuses to participate she is replaced by an alternative person of similar qualifications. However the children associated with the original ML are kept within the study.

In control clusters, no visits will be undertaken (and no micronutrients will be delivered).

Families can refuse any part of the intervention. If severe adverse reactions occur as a result of the sprinkles or if they are refused, they will be discontinued. In the occurrence of diarrhoea, families will be advised to stop micronutrient supplementation temporarily until diarrhoea stops. Low dosage used, consistent with long term administration.

The home visitor will fill in a 'visit form' at the end of each home visit with information on the duration of the visit, participants, activities performed and child progress. 6 women will be hired to supervise home visits throughout the 18 month period. Each supervisor will be allocated to 8 clusters. They will rotate around the 8 clusters regularly, visiting a cluster once every 6-10 weeks. In each cluster, they will spend one week with the 3 home visitors, discussing the child progress in the visits. They will also oversee home visits, and fill in 'monitoring forms' to report on attendance and quality of delivery, etc.

Sprinkles – intake sheets will be distributed that mothers will be asked to fill in to monitor the intake of the child on a daily basis. They will be collected every two weeks when new sprinkles are being delivered to the home. Empty sachets will also be collected.

12: Outcomes

We hypothesise that stimulation will benefit cognitive, language and fine motor development and micronutrients would benefit physical and gross motor development

Primary Outcomes: (1) Children's mental (cognitive), language and motor development

Method of measurement: Bayley Scales of Infant Development; MacArthur-Bates vocabulary check list.
Timepoint: 18 months after the start of treatment
(2) Children’s nutritional status
Method of measurement: Haemoglobin: Hemocue method; Weight: scale (SECA 872); Height: wooden length boards (ShorrBoards), and children’s consumption of iron rich food
Key Secondary Outcomes
Maternal depression
Method of measurement: the 10-item CESD Scale.
2. Investigate the constraints that poor households face when making choices relevant to their children's development, and ultimately to investigate why the intervention works or not. We will do this by building a structural model using detailed socio-economic data at the household level

13: Participant Timeline
The intervention will last 18 months following enrolment. The stimulation component consists of weekly home visits lasting one hour each. For the micronutrient supplementation intervention, sprinkles will be delivered to homes once every two weeks (sufficient for daily intake for all children in the household below 6 years of age).

14: Sample Size
1,440 individuals.

Sample size justification/calculation: we designed the sample to detect a minimum effect of 33% of one standard deviation (SD) of a child development scale (cognitive subscale of the Bayley Scales of Infant Development) for either the micronutrient supplementation only, or the stimulation only intervention against the control group. The level of significance is fixed at 5%, power is fixed at 80%, and the intra cluster correlation at 0.09.

Data on the intra cluster correlation of children’s development scale for rural villages in Colombia are not available. We obtained our estimate of the intra cluster correlation using data from the evaluation of the *Oportunidades* welfare program in Mexico. For this evaluation, various child development tests were collected in rural villages (MacArthur, Peabody, McCarthy, and Woodcock-Johnson). After conditioning on demographic and village variables, the intra cluster correlation was estimated to be between 0.04 and 0.09, depending on the

test. Adopting a conservative approach, we have taken the upper bound of 0.09 for our sample size calculations.

Sample size requirements were computed using "Optimal Design" software (see Raudenbush et al. 2006). An effect of 33% of one SD can be detected at the 5% level of significance and 80% of power with 20 villages per intervention and 21 children per village. The original proposal considered 3 interventions and 6 arms. In the intervention design stage, and given budgetary and logistic restrictions, this was modified to a 2-by-2 design and sample sizes were re-calculated to include 24 villages per intervention and 15 children per village.

15: <u>Recruitment</u>

How potential participants will be identified:

All the participants in the survey will be beneficiaries of the programme Familias en Acción (FeA) and will be recruited by the programme. FeA organises monthly meetings where about 50 beneficiaries meet and discuss education, nutrition and health practices and other activities relevant for the community. In the first of these they will elect a representative who, in the relevant branch of the experiment will be the home visitor. After randomly selecting the groups that will enter the survey, we will contact through the programme, the mothers in the chosen groups who have children aged between 12 and 24 months that attend those meetings.

How potential participants will be approached:

The "madre lider" who liases between the families of the community and the administrator of Familias en Accion will explain the intervention in one of the meetings, and approach the mothers at the end of the meeting to ask them for consent. In the case of the intervention that includes the home visits, through the programme administrators we will explain both the visits and the survey (including the child assessment). In the case of the intervention that includes the micronutrient supplementation, through the programme administrators we will explain both intake of the micronutrients and the survey. In the case of the controls, we will only explain the survey.

How participants will be recruited:

Once contacted, the participants who consent to participate will be included in the survey. The mother will also consent on behalf of her children. Separate consent will be asked to collect the drop of blood sample. The madre lideres that will act as home visitors will be recruited by the programme administrators. If the madre lider of a given group will not want to act as a

home visitor, we will ask her to nominate two substitutes that will be approached to cover that role

Methods: Assignment of interventions (for controlled trials)

16: Allocation

Within each of the 3 regions (each containing 32 municipalities), computer-generated codes will be used to randomly assign eight municipalities to psychosocial stimulation, eight to micronutrient supplementation, eight to both and eight to control. We will use version 9.0 of the Data Analysis and Statistical Software STATA (StataCorp, College Station, TX) to do this.

EF and MRC will together generate the allocation sequence and assign participants to interventions. Interviewers from the Bogota-based data collection company, Sistemas Especializados de Informacion SEI S.A. will enrol participants (by obtaining maternal consent to participate in the survey/child assessment and intervention – if applicable).

17: Blinding

Blinding of study participants to their allocation to stimulation will not be possible and, for ethical reasons, we will not use a placebo for micronutrients, but all efforts will be made to ensure that testers are blind to the treatment condition.

Methods: Data collection, management, analysis

18: Data Collection Methods

Child cognitive, receptive and expressive language, and fine and gross motor development will be assessed using the Bayley Scales of Infant and Toddler Development, Third Edition (Bayley-III) (Bayley 2006), which we will translate into Spanish, back translate, and pilot extensively before use in order to ensure functional and linguistic equivalence. As part of this process, we will compute test-retest reliabilities, aiming for an intra-class correlation of r=0.70 on a minimum of 15 children assessed twice, with a time lapse in between assessments of no less than 5 and no more than 14 days. The Bayley-III will be administered following standard procedures and under controlled conditions. Efforts will be made to test all children under the same conditions in local community centers and in the presence of their mothers. Testers will be psychology graduates and will undertake rigorous training, including practice sessions with children of the target age-groups and from similar socio-economic backgrounds (target is 20 children). The testers will practice

in pairs so that we can collect inter-rater reliabilities. Training/practice testing will continue until $r \geq 0.9$ (intra-class correlation) is obtained per couple of testers and with the trainer.

We will conduct a household survey to the mother/primary caregiver to obtain information on the child, mother and other household members' socio-economic characteristics. These will include age, education level and employment status of all household members, household assets and expenditures. In addition, the household survey will also collect:

(i) maternal depression, using the 10- item Center for Epidemiological Studies Depression (CES-D) Scale,

(ii) maternal time use,

(iii) measures of the quality of the home environment (play materials and play activities) using the Family Care Indicators developed by UNICEF, which is a short version of the HOME,

(iv) measures of child receptive and expressive language, as assessed by maternal report using the Spanish version of the MacArthur-Bates Communicative Development Inventories, Short Form III, (SMBCDI-III) – vocabulary checklist and sentence structure section. At baseline, given the ages of the children, we will administer the vocabulary checklist from SMBCDI-I and SMBCDI-II, to children of 12-18 and 19-24 months of age, respectively. We will pilot the adapted version of the instrument to Colombian Spanish and assess test-retest reliability, as described above (Jackson-Maldonado et al 2003).

(v) Haemoglobin, using the Hemocue method,

(vi) Anthropometric measurements – weight will be measured using a scale (SECA 872) accurate to 0.1 kg, and height will be measured using wooden length boards (ShorrBoards) accurate to 1 mm.

These data will be collected by trained enumerators, who will undertake both theory and practice trainings. For all measurements (i)-(vi) above, we will ensure each enumerator practices with enough children across the target age-range and of similar socio-economic background as the children in the sample. We will collect inter-observer reliabilities as described above.

To avoid any biases, all testers/enumerators will test/interview the same number of children/households in each intervention arm and throughout the entire data collection exercise.

At follow up, the target sample will be the same 1440 children as at baseline. We will aim to re-interview all households, including those that have opted to drop out of the program and those that have migrated to sufficiently nearby areas. We will collect the contact details of 3 relatives/neighbours likely to be able to report the whereabouts of the household in the event of migration. The home visitor/supervisor will be in regular touch with households receiving home visits or micronutrients (or both), which will contribute to minimising attrition.

19: Data Management

Survey data will be collected electronically (using PDAs). Child assessments will be collected on paper and then double-entered and cross-checked to minimise errors. Routine data checks will be carried out to ensure data consistency: any inconsistencies across survey modules or survey questions will be checked against physical questionnaires (if available) or verified with the respondent over the phone. Physical copies of the child assessment report forms will be kept at the headquarters of the data collection company in Bogota until the end of data analysis. Soft copies of the data will be sent over a secured internet portal to the Institute for Fiscal Studies (IFS), where the data will be kept. Any file containing personal information will be encrypted using TrueCrypt, and it will be stored in a folder with access restricted to the investigators of this proposal. Copies of the data security guidelines at IFS are enclosed.

20: Statistical Methods

We will estimate the impact of the intervention using intent-to-treat analyses. Each intervention will be coded with dichotomous variables (yes=1, no=0). More specifically: ‘stimulation’ – stimulation only and both interventions = 1, control and supplementation only = 0; and ‘supplementation’ – supplementation only and both interventions = 1, control and stimulation only = 0; and we will also add an interaction term ‘stimulation*supplementation’ to estimate any synergies from both interventions. We will report mean estimates and 95% confidence intervals for the treatment effects, adjusted for sex, age in months, the baseline level of the outcome (to remove any additional variance), and tester/interviewer. We will cluster standard errors at the cluster (municipality) level.

We will assess balance on observables at baseline by comparing the mean of the primary and secondary outcomes, and other individual, household and

municipality characteristics across intervention groups (clustering standard errors at the cluster (municipality) level).

We hypothesize there may be an interaction of the treatment effect with age. We will test whether the interaction with age is significant and if so we will re-do the analysis by age groups.

In additional statistical analysis (i.e. as robustness checks), we will control for any variables that show imbalances across groups at baseline.

The sample of analysis will be those individuals with non-missing data on the primary outcome of interest (cognitive development) both at baseline and follow up. We will not impute any missing data for primary or secondary outcomes, nor highly relevant covariates such as age and gender of the child. It there is missing or inconsistent data for any of these covariates, we will re-contact the household over the phone to check the information. We will only impute missing data using sample means by region for those covariates that are not crucial for the analysis, such as household assets or maternal education, for example.

Methods: Monitoring

21: Data Monitoring

A data monitoring committee (DMC) will not be established due to minimal risks associated with the study.

There will be no formal interim analyses, as outcome data will only be collected at baseline and follow-up (18 months later).

22: Harms

The PI and other members of the research team (particularly MRC) will be in regular contact with field workers (in particular, with the supervisors of the home visitors) throughout the study, who will report on adverse events and harms. The PI will take the final decision as to whether to discontinue treatment in such events.

23: Auditing

Whilst the research team will design the questionnaires, we will use a fully qualified and well established survey firm in Colombia, Sistemas Especializados de Informacion SEI S.A, to collect the data. It has more than 10 years of experience in conducting interviews in rural Colombia. They

provide extensive training to their staff, including health and safety. Some members of the research team will make regular trips to some of the field areas. In these ways, the quality and completeness of the data will be reflective of the state of the art in clinical trials.

Ethics and Dissemination

24: Research Ethics Approval
Ethics approval will been obtained from the UCL Research Ethics Committee.

25: Protocol Amendments
Any modifications to the protocol which may impact on the conduct of the study, potential benefit of the participant or may affect participant safety, including changes of study objectives, study design, participant population, sample sizes, study procedures, or significant administrative aspects will require a formal amendment to the protocol. Such amendment will be agreed upon by the ESRC and approved by the UCL Ethics Committee prior to implementation.

Administrative changes of the protocol are minor corrections and/or clarifications that have no effect on the way the study is to be conducted.

26: Consent or Assent
Informed consent will be obtained from the child's mother/primary caregiver.

The Hemocue implies finger pricks that are done with sterilized disposable lancets. Separate informed consents will be required from mothers/primary caregivers.

27: Confidentiality
The data will be collected in Colombia and its analysis will be carried out in the UK. The data will be stored at the Institute for Fiscal Studies (IFS). Following recommendation from the UCL Data Officer, the IFS Data Protection Officer has been notified of the project. Copies of the data security guidelines at IFS are enclosed. We will encrypt any file containing personal information using TrueCrypt, and it will be stored in a folder with access restricted to the investigators of this proposal.

The project is compliant with the UK Data Protection Act 1998. There is no Data Protection Registry in Colombia for data collected by private firms.

However, the management systems –including data confidentiality- of the firm that will collect the data has been certified as compliant with international rules 9001 by independent auditors and part of the certification procedure involves the verification of adequate confidentiality protocols for the data collected. We provide copy of the certification. The data will be encrypted using a system with seven security layers.

The project does not require personal data to be sent to Colombia or any other country outside the EU.

28: Declaration of Interests

None of the researchers have any financial relationships with any organisations that might have an interest in the research in the previous three years; nor do they have any other relationships or activities that could appear to have influenced the research.

29: Access to Data

All Investigators will be given access to the cleaned data sets. Project data sets will be housed at the Institute for Fiscal Studies, and all data sets will be password protected. To ensure confidentiality, data dispersed to project team members will be blinded of any identifying participant information.

30: Ancillary and post-trial care

Children who are identified to be severely malnourished, or with serious developmental delays (obvious to the psychologist during the performance of the assessment) will be referred to the health centre.

31: Dissemination Policy

Communicating our results amongst academics will form an integral part of our agenda. Most of the investigators are directly employed in academic institutions and benefit from widespread attendance at national and international conferences on health and development issues. Moreover, they are extensively published in peer-reviewed journals. Communication of our findings to a wide audience of academics will be greatly facilitated by the multi-disciplinary nature of team, which brings together researchers from the fields of economics, psychology and health. Our research will be disseminated through the following channels:

1. Internal seminars at each of the institutions of the research team.
2. International seminars and academic conferences: Members of the research team will travel to world-class universities to present seminars,

and to high-profile international academic conferences to present findings from this research.
3. Published papers: The ultimate output from the research will consist of academic papers published in peer-reviewed journals.

As our research will consider how community-based interventions affect early childhood development, it will also be highly relevant for non-academic users, in particular for policy-makers in developing countries. The operation of our intervention will be closely co-ordinated with the agency that runs the Conditional Cash Transfer program in Colombia. They will receive direct feedback on our activities as we plan to provide them with detailed reports on our results. In addition, our plans for disseminating our findings to policymakers include the following:

1. Communication of our research findings will take place in Colombia primarily during a high-profile conference organised by the research team. Dissemination of our findings in Colombia is greatly assisted by the fact that members of the research team have frequent and ongoing interactions with the research and policy community in Colombia. For example, members of this team regularly spend extended periods in Colombia assisting with field research and making contacts with policy makers, practitioners and the local communities.
2. In disseminating our findings amongst non-academics in the UK, we will benefit from the vast media experience that has been accumulated at the Institute for Fiscal Studies, where many of the co-applicants work. This institution has well-established relationships with various members of the media, and we will communicate our findings to a large public and policy audience by launching events to publicise our results and to ensure that they gain wide coverage in the media.
3. Apart from writing up academic papers, an important part of our dissemination plan will consist of writing up our results in papers that are accessible to policymakers and other non-academic stakeholders such as NGOs.

Authors will be those who make a significant contribution to design, methodology and/or analysis. We intend to list authors in alphabetical order, following convention in the Economics discipline. We do not intend to make use of professional writers.

After completion of the impact evaluation and publication of the main findings from the research, we plan to deposit the (anonymised) data at the

UK Data Archive, as is required of all ESRC-funded projects. At this stage, we plan to make statistical code available to the public.

Appendices

32: Informed Consent Materials

Model consent forms attached (in Spanish).

33: Biological Specimens

We have no plans to collect or store biological specimens for genetic or molecular analysis.

Reference List

Bayley N. Bayley scales of infant and toddler development. 3rd ed. San Antonio (TX): Pearson Education, Inc; 2006.

Engle, P., M. Black, J. Behrman, M. Cabral de Mello, P. Gertler, L. Kapiriri, R. Martorell, and M. Young. 2007. Strategies to avoid the loss of developmental potential in more than 200 million children in the developing world. The Lancet 369 (9557): 229-242.

Grantham-McGregor S, Powell C, Walker S, Himes J. Nutritional supplementation, psychosocial stimulation, and mental development of stunted children: the Jamaican Study. Lancet. 1991;338:1-5.

Grantham-McGregor, Y. Cheung, S. Cueto, P. Glewwe, L. Richter and B. Strupp. 2007. Developmental potential in the first 5 years for children in developing countries. The Lancet 369 (9555): 60 - 70.

Heckman, J. and D. Masterov. 2005. The Productivity Argument for Investing in Young Children. NBER Working Paper Number 13016.

Hoddinott J, Maluccio JA, Behrman JR, Flores R, Martorell R. Effect of a nutrition intervention during early childhood on economic productivity in Guatemalan adults. Lancet. 2008;371:411-6. doi: 10.1016/S0140-6736(08)60205-6.

Jackson-Maldonado D, Thal D, Marchman V, Newton T, Fenson L, Conboy B. MacArthur Inventarios del Desarrollo de Habilidades Comunicativas: user's guide and technical manual. Baltimore: Brookes; 2003.

Lozoff, B., N. Klein, E. Nelson, D. McClish, M. Manuel, and M. Chacon. 1998. Behavior of infants with iron-deficiency anemia. Child Development 69(1):24-36.

Maluccio, J., J. Hoddinott, J. Behrman, R. Martorell, A. Quisumbing, and A. Stein. 2006. The impact of nutrition during early childhood on education among Guatemalan adults. PIER Working Paper No. 06-026.

Martorell, R. 1995. Results and implications of the INCAP follow-up study. Journal of Nutrition 125 (Supplement): 1127S-1138S.

Nelson. C. 2000. The neurobiological basis of early intervention. In Shonkoff JP, Meisels SJ, eds. Handbook of early childhood intervention, 2nd edn. Cambridge: Cambridge University Press.

Raudenbush S. Spybrook J. Liu X-F and R. Congdon. 2006. Optimal Design for Longitudinal and Multilevel Research: Documentation for the Optimal Design Software. [http://sitemaker.umich.edu/group-based/optimal_design_software].

Sen, A. 1999. Development as freedom. New York: Knopf, 1999.

Walker SP, Chang SM, Powell C, Simonoff E, Grantham-McGregor S. Effects of psychosocial stimulation and dietary supplementation in early childhood on psychosocial functioning in late adolescence: follow-up of randomised controlled trial. Br Med J. 2006;333:472.

Walker, S., T. Wachs, J. Meeks Gardner, B. Lozoff, G. Wasserman, E. Pollitt and J. Carter. 2007. Child development: risk factors for adverse outcomes in developing countries. The Lancet 369 (9556): 145-157.

Zlotkin SH, Schauer C, Christofides A, Sharieff W, Tondeur MC, et al. Authors' reply: Sprinkles as a home fortification strategy to improve the quality of complementary foods. PLoS Med 2005;2(7): e202.
