# Supplementary material for: Impacts 2 years after a scalable early childhood development intervention to increase psychosocial stimulation in the home: A follow-up of a cluster randomised controlled trial in Colombia
Source: PLoS Med. 2018 Apr 24;15(4):e1002556. doi: 10.1371/journal.pmed.1002556 (PMC5915272; doi:10.1371/journal.pmed.1002556)
Supplement: S4 Appendix — (PDF) [file pmed.1002556.s005.pdf]

S4 Appendix: 'Scope of Work' from Funding Proposal

The 'scope of work' section from this project's funding proposal is copied below. Points 1 and 2 outline child outcomes.

IV. SCOPE OF WORK

EDePo@IFS will be responsible for the evaluation design, designing the questionnaires, choosing the appropriate developmental tests, overseeing the fieldwork activities, analyzing the collected data so as to estimate the effects of the program on children's development, writing a report on the evaluation findings, and making the data and associated documentation publicly available for researchers.

The above involves, but is not limited to, the following tasks:

1. Design household questionnaires in order to measure socioeconomic characteristics, maternal time use, maternal knowledge regarding parenting practices, the extent of stimulation in the home and parental responsiveness and involvement.

   We propose the **Family Care Indicator (FCI, UNICEF)** and selected subscales (parental involvement, responsiveness and punishment) of the **Home Observations for Measurement of the Environment (HOME)** (Caldwell and Bradley 2001) to collect the latter two. The FCI collects, by direct observation, the number of newspapers, magazines and books for adults in the household; the number of toys the child usually plays with; and by maternal/caregiver report, the number of play activities the child engaged in with an adult over the 3 days before the interview. It has been shown to be a good measure of the level of home stimuli and has been validated against the HOME in a poor environment (Hamadani et al. 2010b).

   In addition we plan to collect, for the first time, novel questions on maternal expectations and maternal beliefs regarding the returns to investments in young children.

2. Identify standardized and well-validated developmental tests to measure the cognitive, language and socio-emotional development of target children. These will be conducted in a centre-based setting and will be chosen in consultation with child development experts and psychologists.

   To measure cognitive development we are considering two candidates: (i) core subtests of the Spanish version of the third edition of the **Wechsler Preschool and Primary Scale of Intelligence Test (WPPSI-III)** (Wechsler 1967; 2002) or (ii) core subtests of the Spanish version of the **Woodcock-Johnson Test of Cognitive Abilities,** the **Batería-III Woodcock-Muñoz: Pruebas de Habilidades Cognitivas** (Woodcock and Johnson 1977; Muñoz-Sandoval et al 2005a,c).

   Both tests are based on direct, in-depth observation of the child's performance in a series of tasks. Tasks vary by age and subtest and must be administered by qualified personnel (e.g.

psychology graduates) who receive extensive training for this purpose. We will work extensively in the design and piloting phases to identify the most appropriate test to use (and appropriate subtests therein).

To measure language development, we are considering to use the Spanish version of the **Peabody Picture Vocabulary Test (PPVT)**, the **Test de Vocabulario en Imágenes de Peabody (TVIP)** (Dunn et al. 1986). The TVIP measures receptive vocabulary from ages 2.5 to 18 years by direct assessment and has been frequently used amongst Latin American populations (Macours et al. 2012; Schady 2011), including Colombia (Bernal and Van Der Werf 2011).

We plan to complement the above with measures of school readiness using the core subtests of the scholastic achievement component of the Woodcock-Muñoz test, the **Batería-III Woodcock-Muñoz: Pruebas de Aprovechamiento** (Muñoz-Sandoval et al 2005a,b) or a similar test.

To measure socio-emotional development, we plan to use the **Strengths and Difficulties Questionnaire (SDQ)**, a behavioral screening questionnaire consisting of 25 items for completion by the primary caregivers of 4-16 years old (Goodman 1997). It is quick and relatively easy to administer and has been widely used in developing countries (it is available in Spanish). We also propose to measure attention span and executive functioning, possibly using the short version of the attention focusing and inhibitory control subscales of the widely used **Children's Behavior Questionnaire** (CBQ; Rothbart et al. 2001; Putnam and Rothbart 2006), designed to measure temperament in children aged 3-7 years.

3. To choose appropriate home-based tests to measure the cognitive and linguistic development of the next younger sibling of the target child.

   We propose to assess the development of younger sibling of the target children using the **Ages and Stages Questionnaire (ASQ-3)** (Squires and Bricker 2009; Squires et al. 1997) and complement it with a measure of receptive and expressive language as per maternal report using the Spanish versions of the **MacArthur-Bates Communicative Development Inventories (CDI)** (Fenson et al. 2000; Jackson-Maldonado et al. 2003).

   The ASQ-3 measures skills in five key developmental areas—communication, gross motor, fine motor, problem solving and personal-social—on the basis of maternal reports and observations for children 0-60 months old. Administration times are 15-20 minutes on average.

   The CDI inventories have been shown to provide a valid assessment of language skills for Spanish speaking children 8 to 42 months (split into three inventories for different age ranges) and have been linked with important biological outcomes (Marchman and Martínez-Sussmann, 2002) and later development (Hamadani et al. 2010a).

   We plan that both the ASQ-3 and the CDI forms will be administered in the home as part of the household survey by the (trained) interviewer.

4. To adapt to the context and pilot the psychometric tests in points 2. and 3. above. This will be done in close collaboration with Prof Grantham-McGregor and a local psychologist(s) as described in the next section.

5. To coordinate the preparation of data collection activities, including the training of teams of interviewers and testers in the administration of the household survey and the psychometric tests in points 2. and 3. above. Training will include practices with at least 20 children of the appropriate age range and socio-economic level living in or near Bogota.

6. The support the Colombian survey firm and project coordinator in advance of and throughout the data collection period.

7. To prepare a clean, ready to use data set, and associated documentation, and to make them publicly available to researchers.

8. To analyze the data using standard statistical methods and software and to write up evaluation findings in the form of both policy reports and academic papers.

9. To organize and participate in a conference in Bogota in the first half of 2014, to launch the findings of the medium term evaluation, and to travel to international conferences to disseminate findings of the research study.

10. To ensure the academic integrity of the data collection and data evaluation processes are maintained at all times.
