# Supplementary material for: Impacts 2 years after a scalable early childhood development intervention to increase psychosocial stimulation in the home: A follow-up of a cluster randomised controlled trial in Colombia
Source: PLoS Med. 2018 Apr 24;15(4):e1002556. doi: 10.1371/journal.pmed.1002556 (PMC5915272; doi:10.1371/journal.pmed.1002556)
Supplement: S5 Appendix — (PDF) [file pmed.1002556.s006.pdf]

S5 Appendix: Construction of Scores Using IRT and GRM

As detailed in S1 Table we scored all measures of child development and the quality of the home environment with item level data available using either a 2-parameter Item Response Theory (IRT) model or a Graded Response Model (GRM). We used IRT when item level data was binary and GRM when it was ordered but discrete.

In the 2-parameter IRT model, whether or not a child *m* correctly answers item *i* is a function of the difficulty of that item (α_i), its discrimination parameter(β_i) and the child's underlying ability in that domain (θ_m) which is assumed to follow a standard normal distribution with zero mean and unit variance. Specifically:

$$x_{im} = 1 \text{ if } x *_{im} > 0$$

$$x_{im} = 0 \text{ otherwise}$$

$$x *_{im} = \alpha_i + \beta_i \, \theta_m + \epsilon_{im}$$

where $x_{im} = 1$ denotes child *m* answering item *i* correctly. ϵ_{im} is assumed to follow a type-I extreme value distribution and conditional on θ_m all the ϵ_{im} are independent. Therefore, the probability of child *m* answering item *i* correctly is:

$$\Pr(x_{im} = 1 \mid \theta_m) = \frac{\exp\{\alpha_i + \beta_i \, \theta_m\}}{1+\exp\{\alpha_i + \beta_i \, \theta_m\}}, \; \theta_m \sim N(0,1)$$

The Graded Response Model that we used for ordered item data (e.g. where a child can get a 0, 1, 2 or 3 for each item) is very similar to the IRT model above except now there are as many difficulty parameters, or cut-offs, as the number of different scores a child can obtain on that item. Therefore, the probability that child *m* got a score of at least *k* on item *i* is:

$$\Pr(x_{im} \geq k \mid \theta_m) = \frac{\exp\{\alpha_{ik} + \beta_i \, \theta_m\}}{1+\exp\{\alpha_{ik} + \beta_i \, \theta_m\}}, \; \theta_m \sim N(0,1)$$

We estimated item level difficulty and discrimination parameters using STATA's IRT command. We used mean and variance adaptive Gauss-Hermite quadrature with 21 quadrature points to integrate out θ_m when estimating parameters. We then used Empirical Bayes methods to predict each child's underlying θ_m and the standard error of this prediction.

To ensure parameter estimates were stable and meaningful we only used items attempted by at least 20% of the sample.
