# Supplementary material for: Impacts 2 years after a scalable early childhood development intervention to increase psychosocial stimulation in the home: A follow-up of a cluster randomised controlled trial in Colombia
Source: PLoS Med. 2018 Apr 24;15(4):e1002556. doi: 10.1371/journal.pmed.1002556 (PMC5915272; doi:10.1371/journal.pmed.1002556)
Supplement: S6 Appendix — (PDF) [file pmed.1002556.s007.pdf]

S6 Appendix: Validation of Child Development Measures

Tables A and B present basic validation tests for the measures of cognitive, language, school readiness, executive functioning, and behavioural development.

For all measures scored using IRT/GRM we estimated the reliability of each measure for each child, i.e. the proportion of variance in the observed score that is due to variance in the true underlying construct that instrument is measuring. This is equal to:

$$reliability(\theta_m) = \frac{\frac{1}{se(\theta_m)^2}}{1 + \frac{1}{se(\theta_m)^2}}$$

where $se(\theta_m)$ is the estimated standard error on our prediction of θ_m from the empirical Bayes procedure. In tables A and B we report the mean reliability across the sample.

We also assessed the validity of our measures by assessing how they correlate with each other (Tables C) and with previous measures of child development—namely the Bayley-III two years earlier, with age, and with socio-economic characteristics (Tables A and B).

Except concept formation (Woodcock-Munoz Cognitive 5), all other measures of language, school readiness, executive functioning had estimated reliabilities of at least 0.7, for scores constructed using IRT/GRM. They also correlated with each other, age, lagged measures of child development (Bayley-III scores), wealth and maternal education in the expected direction. Woodcock-Munoz Cognitive 5 has a very low estimated reliability of 0.371 and displays low correlation with relevant variables. This is due to the majority of children (73%) not scoring enough in the first five questions to proceed to later items so there is little information available. On the basis of this very low estimated reliability, and inconsistent correlations with other measures and socio-economic variables, we exclude this measure from the analysis.

All subscales of the SDQ and the CBQ correlate highly significantly ($p<0.01$) in the expected direction. Unlike the previous measures, these show no correlation with age. They do correlate in the expected direction with measures of behavioural development from endline (30-42 months), with the strongest correlations being observed for subscales measuring the same construct, and with wealth and maternal education.

		Correlation coefficient of Child Development measure[2] with								
			Bayley Scores at Endline (30-42 months)[3]							
	Mean reliability[1]	Age	Cognition	Receptive Language	Expressive Language	Fine Motor	Gross Motor	Wealth index[4]	Mother's education[5]	N
**Instruments scored using IRT or GRM model**										
WM Concept Formation	0.37	0.18***	0.17***	0.22***	0.16***	0.20***	0.08**	0.09**	0.13***	1,251
WM Picture Recognition	0.70	0.21***	0.31***	0.32***	0.29***	0.26***	0.16***	0.13***	0.12***	1,254
WM Memory for Names	0.85	0.27***	0.35***	0.36***	0.32***	0.31***	0.18***	0.19***	0.21***	1,252
WM Expressive Language	0.82	0.23***	0.43***	0.43***	0.39***	0.33***	0.20***	0.19***	0.21***	1,255
TVIP (Receptive Language)	0.88	0.36***	0.47***	0.48***	0.43***	0.40***	0.19***	0.26***	0.25***	1,253
Daberon-2 (School Readiness)	0.94	0.43***	0.53***	0.52***	0.51***	0.48***	0.31***	0.21***	0.24***	1,255
**Non-IRT Measures[7]**										
WM Visual Matching		0.32***	0.38***	0.33***	0.29***	0.36***	0.18***	0.14***	0.16***	1,251
WM Retrieval Fluency		0.25***	0.25***	0.29***	0.33***	0.21***	0.19***	0.10***	0.09**	1,251
WM Decision Speed		0.29***	0.38***	0.34***	0.26***	0.38***	0.22***	0.15***	0.21***	1,252
PTT (Inhibitory Control and Working Memory)		0.29***	0.27***	0.30***	0.24***	0.35***	0.20***	0.11***	0.14***	1,251

Table A: Validity of measures of Child Cognition, Language, School Readiness, and Executive Function (measures directly assessed in a centre by a psychologist). Correlation coefficient significantly different from zero at: * $p < 0.05$, ** $p < 0.01$, *** $p < 0.001$. [1]Mean reliability as calculated and defined above. [2]For the correlation coefficient of the measures with age we use the IRT/GRM (or count) score before non-parametric standardization, for all other correlation coefficients we use the IRT (or count) score after non-parametric standardization (removing all age effects). [3]We use non-parametrically standardized Bayley scores from endline (removing all age effects). [4]Wealth index constructed using factor analysis of baseline asset ownership. [5]Years of education of mother as measured at baseline. [6]Refers to measures which we constructed using IRT/GRM models. [7]Refers to measures which are count measures and therefore are not suited for scoring using Item Response Theory.

	Mean reliability[1]	Correlation coefficient of Child Behavioural Development measure[1] with										
			Bates Scores at Endline (30-42 months) [2]				Rothbart Scores at Endline (30-42 months) [2]					
		Age	Difficult†	Unstoppable†	Unadaptable†	Unsociable†	Attention	Inhibit	Sociable	Wealth index[3]	Mother's education[4]	N
SDQ Hyperactivity†	0.65	0.09**	0.18***	0.20***	0.02	0.09**	-0.13***	-0.23***	-0.07*	-0.06*	-0.11***	1,250
SDQ Emotional Symptoms†	0.70	0.02	0.16***	0.05	0.14***	0.05	-0.06*	-0.12***	-0.06*	-0.08**	-0.05	1,250
SDQ Conduct Problems†	0.71	0.05	0.30***	0.24***	-0.01	0.08**	-0.13***	-0.27***	-0.03	-0.08**	-0.04	1,250
SDQ Peer Problems†	0.67	0.07**	0.14***	0.09**	0.13***	0.14***	-0.07*	-0.11***	-0.18***	-0.13***	-0.11***	1,250
SDQ Total Difficulties†, [5]	0.70	0.01	0.31***	0.23***	0.11***	0.14***	-0.14***	-0.29***	-0.12***	-0.14***	-0.12***	1,250
SDQ Prosocial Behaviour	0.68	-0.02	-0.15***	-0.08**	-0.05	-0.19***	0.14***	0.15***	0.22***	0.12***	0.09**	1,250
CBQ Attention Focusing	0.81	0.00	-0.28***	-0.27***	-0.11***	-0.16***	0.21***	0.34***	0.10***	0.09**	0.14***	1,250
CBQ Inhibitory Control	0.75	-0.04	-0.16***	-0.19***	-0.01	-0.16***	0.20***	0.18***	0.11***	0.09**	0.17***	1,250

Table B: Validity of measures of Child Behaviour (measures collected in the home by parental report). Correlation coefficient significantly different from zero at: $^{*}\ p < 0.05$, $^{**}\ p < 0.01$, $^{***}\ p < 0.001$. [1]For the correlation coefficient of the measures with age we use the score before non-parametric standardization, for all other correlation coefficients we use the score after non-parametric standardization (removing all age effects). [2]We use non-parametrically standardized Bates and Rothbart scores from endline (removing all age effects). [3]Wealth index constructed using factor analysis of baseline asset ownership. [4]Years of education of mother as measured at baseline. †Measure scored such that higher values indicate more problems/lower levels of behavioural development. [5]Total Difficulties subscale added following peer review, it contains items from four SDQ subscales (Hyperactivity, Emotional Symptoms, Conduct Problems, Peer Problems).

	Measures of cognition, language, school readiness and executive functioning, directly assessed in a centre by a psychologist										Measures of behavioural development, collected in the home by parental report (interviewer)						
	WM Concept Formation	WM Visual Matching	WM Retrieval Fluency	WM Concept Formation	WM Decision Speed	WM Memory for Names	WM Expressive Language	TVIP (Receptive Language)	Daberon-2 (School Readiness)	PTT (Inhibitory Control and Working Memory)	SDQ Hyperactivity	SDQ Emotional Symptoms	SDQ Conduct Problems	SDQ Peer Problems	SDQ Prosocial Behaviour	CBQ Attention Focusing	CBQ Inhibitory Control
WM Concept Formation	1																
WM Visual Matching	0.23***	1															
WM Retrieval Fluency	0.13***	0.28***	1														
WM Picture Recognition	0.21***	0.34***	0.17***	1													
WM Decision Speed	0.22***	0.45***	0.22***	0.30***	1												
WM Memory for Names	0.22***	0.38***	0.24***	0.39***	0.37***	1											
WM Expressive Language	0.26***	0.38***	0.33***	0.35***	0.33***	0.46***	1										
TVIP (Receptive Language)	0.29***	0.47***	0.31***	0.42***	0.44***	0.50***	0.68***	1									
Daberon-2	0.31***	0.55***	0.39***	0.41***	0.47***	0.45***	0.60***	0.66***	1								
PTT	0.26***	0.33***	0.16***	0.25***	0.32***	0.31***	0.28***	0.34***	0.43***	1							
SDQ Hyperactivity†	-0.08**	-0.15***	-0.13***	-0.07*	-0.16***	-0.12***	-0.09**	-0.12***	-0.18***	-0.13***	1						
SDQ Emotional Symptoms†	-0.02	-0.08**	-0.06*	-0.06*	-0.07*	-0.02	-0.10***	-0.09**	-0.10***	-0.03	0.18***	1					
SDQ Conduct Problems†	-0.08**	-0.15***	-0.07*	-0.08**	-0.14***	-0.14***	-0.11***	-0.15***	-0.17***	-0.12***	0.37***	0.24***	1				
SDQ Peer Problems†	-0.05	-0.10***	-0.06*	-0.12***	-0.09**	-0.09**	-0.10***	-0.16***	-0.16***	-0.07*	0.09**	0.24***	0.16***	1			
SDQ Prosocial Behaviour	0.05	0.12***	0.09**	0.05	0.12***	0.09**	0.11***	0.16***	0.18***	0.07*	-0.26***	-0.10***	-0.27***	-0.20***	1		
CBQ Attention Focusing	0.05	0.14***	0.10***	0.08**	0.17***	0.15***	0.10***	0.18***	0.21***	0.13***	-0.44***	-0.12***	-0.47***	-0.12***	0.34***	1	
CBQ Inhibitory Control	0.04	0.16***	0.09**	0.10***	0.16***	0.16***	0.11***	0.14***	0.20***	0.15***	-0.52***	-0.15***	-0.36***	-0.13***	0.32***	0.52***	1

Table C: Contemporaneous Correlation Between all Measures of Child Development. Correlation coefficient significantly different from zero at: * $p < 0.05$, ** $p < 0.01$, *** $p < 0.001$. All measures are non-parametrically standardized to remove age effects. †Measure scored such that higher values indicate more problems/lower levels of behavioural development.
