# Supplementary material for: Impacts 2 years after a scalable early childhood development intervention to increase psychosocial stimulation in the home: A follow-up of a cluster randomised controlled trial in Colombia
Source: PLoS Med. 2018 Apr 24;15(4):e1002556. doi: 10.1371/journal.pmed.1002556 (PMC5915272; doi:10.1371/journal.pmed.1002556)
Supplement: S7 Appendix — (PDF) [file pmed.1002556.s008.pdf]

S7 Appendix: Comparing Short-Term and Medium-Term Effect Sizes

We tested whether the medium-term effects were significantly different from the short-term effects. This test assesses whether null results obtained at the 2 year follow-up are driven by increased noise in the outcome measures. In making this comparison it is important that the short-term and medium-term impacts are measured in the same unit. We scale both outcome variables to have a mean of zero and a standard deviation (SD) of one in the control group. Hence both effect sizes can be interpreted relative to the SD of the control group and the magnitudes are directly comparable.

The short-term and medium-term outcome measures are from different child development assessments: Bayley-III cognitive and receptive language scores in the short-term and a factor score derived from 9 assessments related to cognition, language, school readiness and executive function in the medium-term. However, since both are age-appropriate measures of cognition and language, scaled identically, we consider this a useful comparison.

To assess the statistical significance of the difference requires estimating the covariance between estimators for the short-term and medium-term effects. In the short-term (ST) evaluation and medium-term (MT) evaluation we separately estimated effects by OLS using the following estimating equations:

$$y_j = \beta_j T + \gamma_j X_j + \varepsilon_j \text{ for } j = ST, MT$$

where y_j is the relevant outcome variable, T is the treatment indicator, X_j are controls and ε_j is the error term.

We can test the difference in magnitudes between the short-term and medium-term effects by estimating the two sets of coefficients jointly on 'long' data where the outcome variable y takes the value of the short-term outcome y_{ST} if $j = ST$ and y_{MT} if $j = MT$. The relevant estimating equation is:

$$y = \beta_{ST} T * I_{ST} + \gamma_{ST} X_{ST} * I_{ST} \quad + \beta_{MT} T * I_{MT} + \gamma_{MT} X_{MT} * I_{MT} \quad + \varepsilon$$

where $I_{ST} = 1$ if $j = ST$ and $I_{MT} = 1$ if $j = MT$. Mechanically, the estimates of all coefficients are identical to when the short-term and medium-term impacts are estimated separately. However, one obtains the joint covariance matrix enabling us to test the null hypothesis: $\beta_{ST} = \beta_{MT}$

In practice, we use the *suest* procedure on STATA to estimate this specification and the joint covariance matrix.

Table D displays estimated differences between the short-term and medium-term effects on child cognitive and language development:

	Stimulation	Supplementation	Stimulation and Supplementation	n
**Short-Term Cognitive Effect Size (Bayley-III Cognition) vs. Medium-Term Cognitive Effect Size (Cognitive Factor)**				
Short-Term	0.27***<br>(0.10 to 0.43)	-0.05<br>(-0.21 to 0.11)	0.25***<br>(0.10 to 0.40)	1153
Medium-Term	-0.03<br>(-0.23 to 0.16)	-0.04<br>(-0.25 to 0.16)	-0.11<br>(-0.31 to 0.09)	1243
Difference (Short-Term – Medium-Term)	0.30***<br>(0.12 to 0.47)	-0.005<br>(-0.18 to 0.17)	0.36***<br>(0.16 to 0.57)	
**Short-Term Receptive Language Effect (Bayley-III Language) vs. Medium-Term Cognitive Effect (Cognitive Factor)**				
Short-Term	0.21**<br>(0.02 to 0.40)	-0.01<br>(-0.22 to 0.19)	0.13<br>(-0.04 to 0.30)	1153
Medium-Term	-0.03<br>(-0.23 to 0.16)	-0.04<br>(-0.25 to 0.16)	-0.11<br>(-0.31 to 0.09)	1243
Difference (Short-Term – Medium-Term)	0.24**<br>(0.04 to 0.43)	0.030<br>(-0.17 to 0.23)	0.24**<br>(0.01 to 0.46)	

Table D: Difference Between Medium-Term and Short-Term Effect Sizes. *p<0.1, **p<0.05, ***p<0.01: 2-tailed p-values for testing null hypothesis that coefficient is equal to zero. 95% CIs (in parentheses) and P-values adjusted for clustering at the town level. All scores (short-term and medium-term) standardized non-parametrically with respect to age and have zero mean and unit variance in the control group. Estimates control for baseline levels of cognitive, receptive language, expressive language, fine motor and gross motor development, as assessed by the Bayley-III; children's sex; and tester dummies. Short-Term effects estimated for the sub-sample for whom the cognitive factor is defined at the medium-term follow-up. Differences calculated and tested using STATA's suest command.
