# Supplementary material for: Impacts 2 years after a scalable early childhood development intervention to increase psychosocial stimulation in the home: A follow-up of a cluster randomised controlled trial in Colombia
Source: PLoS Med. 2018 Apr 24;15(4):e1002556. doi: 10.1371/journal.pmed.1002556 (PMC5915272; doi:10.1371/journal.pmed.1002556)
Supplement: S1 Table — (PDF) [file pmed.1002556.s009.pdf]

Domain	Function assessed	Instrument	Scoring Method
**A. Primary Outcome: Child Development**			
**Measures of cognition, language, school readiness and executive functioning, directly assessed in a centre by a psychologist**			
Cognition	Fluid Reasoning	WM Cognitive 5: Concept Formation	IRT
	Processing Speed	WM Cognitive 6: Visual Matching	NC
	Long-term Retrieval	WM Cognitive 12: Retrieval Fluency	NC
	Visual-Spatial Thinking	WM Cognitive 13: Picture Recognition	GRM
	Processing Speed	WM Cognitive 16: Decision Speed	NC
	Short Term Memory	WM Cognitive 21: Memory for Names	IRT
Language	Expressive Language	WM Achievement 14: Picture Vocabulary	IRT
	Receptive Language	TVIP	IRT
School Readiness	School Readiness	Daberon-2	IRT
Executive Functioning	Inhibitory Control and Working Memory	Pencil Tapping Task	NC
**Measures of children's behaviour, collected in the home by parental report (interviewer)**			
Behaviour	Hyperactivity†	SDQ Hyperactivity	GRM
	Emotional Symptoms†	SDQ Emotional Symptoms	GRM
	Conduct Problems†	SDQ Conduct Problems	GRM
	Peer Problems†	SDQ Peer Problems	GRM
	Prosocial Behaviour	SDQ Prosocial	GRM
	Total Difficulties[1]	SDQ Total Difficulties	GRM
	Attentional Focusing	CBQ Attentional Focusing	GRM
	Inhibitory Control	CBQ Inhibitory Control	GRM
**B. Secondary Outcomes: Stimulation in the Home Environment**			
	Variety of Play Activities	FCI Play Activities	GRM
	Variety of Play Materials	FCI Play Materials	GRM
	Maternal Depressive Symptoms	CES-D 10	GRM

S1 Table: Measures of Primary and Secondary Outcomes. Columns 1 and 2 present the domain and function assessed, as given in the test manual. Column 3 presents the instrument used. Column 4 presents the method used to score the instrument: IRT =2-parameter Item Response Theory model, GRM=Graded Response Model, NC=Number Correct. WM =Woodcock-Muñoz, TVIP =Test de Vocabulario en Imágenes de Peabody, SDQ =Strengths and Difficulties Questionnaire, CBQ =Child Behaviour Questionnaire, FCI =Family Care Indicators, CES-D 10 = 10-item Center for Epidemiologic Studies short depression scale. †Measure scored such that higher values indicate more problems/lower levels of behavioural development. [1]Total Difficulties subscale contains items from SDQ subscales: Hyperactivity, Emotional Symptoms, Conduct Problems and Peer Problems.
