# Supplementary material for: Impacts 2 years after a scalable early childhood development intervention to increase psychosocial stimulation in the home: A follow-up of a cluster randomised controlled trial in Colombia
Source: PLoS Med. 2018 Apr 24;15(4):e1002556. doi: 10.1371/journal.pmed.1002556 (PMC5915272; doi:10.1371/journal.pmed.1002556)
Supplement: S2 Table — (PDF) [file pmed.1002556.s010.pdf]

	Attrition at 2 year follow-up	
**Stimulation**	0.02	0.01
	(-0.03 to 0.07)	(-0.04 to 0.06)
**Supplementation**	0.04	0.03
	(-0.02 to 0.09)	(-0.02 to 0.09)
**Stimulation and Supplementation**	0.05*	0.06*
	(-0.01 to 0.11)	(-0.00 to 0.11)
**Baseline Age in Months**		0.00
		(-0.00 to 0.00)
**Baseline Cognition (Bayley-III)**		0.01
		(-0.01 to 0.03)
**Baseline Receptive Language (Bayley-III)**		-0.02*
		(-0.03 to 0.00)
**Baseline Expressive Language (Bayley-III)**		-0.00
		(-0.02 to 0.02)
**Baseline Fine Motor (Bayley-III)**		0.00
		(-0.01 to 0.02)
**Baseline Gross Motor (Bayley-III)**		-0.00
		(-0.02 to 0.02)
**Male**		-0.05***
		(-0.08 to -0.01)
**Constant**	0.09***	0.11**
	(0.05 to 0.13)	(0.02 to 0.20)
**Observations**	1,419	1,417
**R-squared**	0.004	0.011

S2 Table: Differential Attrition by Treatment Status and Baseline Characteristics. Table displays results from an OLS regression of a binary indicator =1 if the child attritted from the sample at this follow-up on treatment indicators, baseline characteristics and a constant term. *p<0.10, **p<0.05, ***p<0.01: 2-tailed p-values for difference of regression coefficient from zero. 95% CIs (in parentheses) and P-values adjusted for clustering at the town level. Baseline Bayley-III scores standardised non-parametrically for age and scaled to have zero mean and unit variance in the control group.
