# Supplementary material for: Impacts 2 years after a scalable early childhood development intervention to increase psychosocial stimulation in the home: A follow-up of a cluster randomised controlled trial in Colombia
Source: PLoS Med. 2018 Apr 24;15(4):e1002556. doi: 10.1371/journal.pmed.1002556 (PMC5915272; doi:10.1371/journal.pmed.1002556)
Supplement: S3 Table — (PDF) [file pmed.1002556.s011.pdf]

Domain	Instrument	Factor 1 (rotated) Loading	Factor 2 (rotated) Loading
**Cognition**	WM Visual Matching	0.62	0.12
	WM Retrieval Fluency	0.40	0.08
	WM Picture Recognition	0.52	0.03
	WM Decision Speed	0.56	0.14
	WM Memory for Names	0.61	0.09
**Language**	WM Expressive Language	0.71	0.03
	TVIP (Receptive Language)	0.81	0.08
**School Readiness**	Daberon-2 (School Readiness)	0.82	0.16
**Executive Functioning**	PTT (Inhibitory Control and Working Memory)	0.46	0.11
**Behaviour**	SDQ Hyperactivity†	-0.10	-0.64
	SDQ Emotional Symptoms†	-0.08	-0.25
	SDQ Conduct Problems†	-0.12	-0.58
	SDQ Peer Problems†	-0.15	-0.21
	SDQ Prosocial Behaviour	0.12	0.44
	CBQ Attention Focusing	0.12	0.71
	CBQ Inhibitory Control	0.12	0.70
**Eigenvalue**		**4.081**	**1.679**

Table S3: Exploratory Factor Analysis of Child Development Measures. Exploratory factor analysis. 2 factors retained and rotated using Kaiser criterion. †Measure scored such that higher values indicate more problems/lower levels of behavioural development. Scoring of all measures outlined in S1 Table.
