# Supplementary material for: Impacts 2 years after a scalable early childhood development intervention to increase psychosocial stimulation in the home: A follow-up of a cluster randomised controlled trial in Colombia
Source: PLoS Med. 2018 Apr 24;15(4):e1002556. doi: 10.1371/journal.pmed.1002556 (PMC5915272; doi:10.1371/journal.pmed.1002556)
Supplement: S4 Table — (PDF) [file pmed.1002556.s012.pdf]

	Cognitive Factor	Behavioural Factor
**=1 if Maternal Education 11 or more years**	0.473***	0.188***
	(0.355 to 0.590)	(0.0593 to 0.317)
**Constant**	-0.177***	-0.0396
	(-0.269 to -0.0851)	(-0.124 to 0.0443)
**Observations**	1,243	1,243
**R-squared**	0.050	0.007

Table S4: Child Development by Maternal Education. Table displays results from OLS regressions of primary outcomes measures (cognitive development factor and behavioural development factor) on indicators for maternal education. The excluded group (captured by the constant term) is children with mothers who have 11 or fewer years of education. *p<0.10, **p<0.05, ***p<0.01: 2-tailed p-values for difference of regression coefficient from zero. 95% CIs (in parentheses) and P-values adjusted for clustering at the town level.
